# Computer Model Based on an Asynchronous BLE 5.0 IMU Sensor Network for Biomechanical Applications

**DOI:** 10.3390/s25237271

**Published:** 2025-11-28

**Authors:** Juan Antonio Mora-Sánchez, Luis Pastor Sánchez-Fernández, Diana Lizet González-Baldovinos, María Teresa Zagaceta-Álvarez, Sandra Dinora Orantes-Jiménez

**Affiliations:** 1 Centro de Investigación en Computación, Instituto Politécnico Nacional, Av. Juan de Dios Bátiz S/N, Nueva Industrial Vallejo, Gustavo A. Madero, Mexico City 07738, Mexico; jmoras1400@alumno.ipn.mx (J.A.M.-S.); d.gonzalezb@cic.ipn.mx (D.L.G.-B.); dinora@cic.ipn.mx (S.D.O.-J.); 2 Escuela Superior de Ingeniería Mecánica y Eléctrica Unidad Azcapotzalco, Instituto Politécnico Nacional, Av. de las Granjas 682, Azcapotzalco, Mexico City 02250, Mexico; mzagaceta@ipn.mx

**Keywords:** asynchronous data acquisition, Bluetooth Low Energy 5.0, fuzzy inference system, IMUs, rapid upper limb assessment

## Abstract

**Highlights:**

**What are the main findings?**

- Inertial Measurement Unit network using a hybrid asynchronous architecture with real-time data acquisition.
- BLE 5.0 communication with a net throughput of 1.4 Mbps and a range of 105 m.
- Fuzzy inference models using the RULA method, converting biomechanical data into a risk of injury score.
- Models that quantify the temporal distribution of injury risk.

**What is the implication of the main finding?**

- The validated BLE 5.0 asynchronous network provides the robustness and scalability needed to replace restrictive, high-cost systems, enabling reliable data acquisition for complex motion analysis.
- The fuzzy RULA model addresses subjectivity and discretization issues in the traditional approach, offering an objective, higher-resolution metric suitable for real-time monitoring and informed decision-making.
- Quantifying exposure time provides a key metric for occupational health, enabling the deployment of personalized interventions and data-driven preventive strategies.

**Abstract:**

The acquisition, processing, and monitoring of biomechanical variables in dynamic environments require sensor network architectures capable of handling high concurrency and large data volumes. This study aims to develop, validate, and deploy a robust asynchronous network architecture of Inertial Measurement Units (IMUs) utilizing Bluetooth Low Energy (BLE) 5.0 for real-time biomechanical signal acquisition, overcoming the range, speed, and stability limitations of prior implementations. A network of six IMUs was implemented, with communication managed by a hybrid Python 3.10–LabVIEW 2022 Q3 framework. This architecture ensures concurrent, asynchronous data acquisition while maintaining stable sensor interconnection through virtual port emulation. System evaluation demonstrated superior technical performance, exhibiting high acquisition efficiency (close to 100%) and data loss below ±2% across 75 assessments per sensor. These assessments were obtained by evaluating the posture of 25 participants during three postural experiments, with a maximum indoor range of 40 m and an outdoor range of 105 m, validating the system’s scalability and robustness for motion capture. The approach was applied in a case study using a Fuzzy Inference System (FIS) to assess the upper limb via the Rapid Upper Limb Assessment (RULA) method. The system successfully quantified the temporal distribution of injury risk bilaterally, overcoming the limitations of observational methods and providing objective metrics crucial for occupational health in seated tasks.

## 1. Introduction

Technological advances in Inertial Measurement Units (IMUs) have evolved remarkably—from the mechanical gyroscopes used in aerospace systems in the 1940s–1960s, to strapdown systems in the 1970s–1980s and, subsequently, to microelectromechanical system (MEMS)-based sensors from the late 1980s onward [1,2]. These developments have enabled the creation of increasingly compact, cost-effective, and energy-efficient devices, expanding their application beyond the aerospace domain to a wide range of scientific and clinical fields. In biomechanics, IMUs have become essential tools for quantifying human movement, analyzing gait, and evaluating physical performance, offering a portable and non-invasive alternative to traditional optical motion-capture systems. Owing to their ability to record acceleration and angular velocity with high temporal resolution, IMUs represent a key enabling technology for emerging applications in rehabilitation, movement control, and sports performance monitoring [2].

IMUs operate based on Newton’s laws of motion, enabling the detection of acceleration, angular velocity, and related kinematic variables by converting physical signals into electrical outputs. These devices typically integrate accelerometers and gyroscopes—which measure linear acceleration and angular velocity, respectively—and provide measurements around three orthogonal axes (yaw, pitch, and roll), corresponding to rotations about the X, Y, and Z coordinates of the object being monitored. Their advantages include low cost, customizability, ease of integration, and user comfort [1].

Inertial sensors support a broad range of case applications, enabling the estimation of position, velocity, acceleration, and other biomechanical or motion-related variables in both humans and objects. They are widely used in domains such as virtual and augmented reality, navigation, and human-movement analysis [3]. Among the primary approaches for estimating these variables—optical motion-capture systems, camera-based image tracking, and IMUs—the latter offers the most cost-effective and scalable solution. IMUs are particularly advantageous in multi-device configurations, providing reliable performance, reduced complexity, and flexibility in tracking multiple sensors or subjects simultaneously. Their effectiveness has been consistently demonstrated in applications involving both healthy individuals and clinical populations, including the quantification of motor signals in control subjects and patients with Parkinson’s disease [4,5,6,7].

Some current inertial sensor-based motion capture systems rely on Wi-Fi or other radio-frequency protocols that depend on a central access point. Because all data is routed through this single node, direct interaction between nearby devices is limited and system scalability is restricted [3]. Although various commercial solutions exist for estimating human motion [8,9,10]—primarily for animation, healthcare, and sports—they often use proprietary interfaces that decrease interoperability, as data collection and processing require manufacturer-specific receivers and software.

Bluetooth technology has become a crucial enabler for short-range wireless communication in Internet of Things (IoT) systems. Earlier Bluetooth versions (2.0 and 3.0) were designed for large data packets and high throughput, while the introduction of Bluetooth 4.0 in 2011 marked the first implementation of Bluetooth Low Energy (BLE) [11]. BLE significantly reduces energy consumption while maintaining sufficient performance and low latency, allowing efficient point-to-point communication without extra infrastructure [3,12]. The improvements in BLE 5.0—including higher data rates, extended range, and support for multiple devices at once—have established it as the standard for modern biomedical and industrial wireless systems [13]. Additionally, BLE 5.0 has shown high energy efficiency even in densely populated device environments [14].

Recent research on wireless sensor networks for lower-limb rehabilitation has shown that BLE 5.0-based asynchronous communication can support multi-IMU configurations (up to four devices) with stable transmission rates and real-time visualization in LabVIEW; however, some data loss and limited synchronization were observed during fast movements [15]. Other studies have explored IMU-BLE architectures for large-scale applications, yet their scalability remains constrained by dependency on mobile infrastructure and increased battery consumption [16]. Alternative designs, such as asynchronous BLE 5.1 networks integrating IMUs with electromyography (EMG) sensors, have demonstrated successful miniaturization but at the cost of reduced sampling frequency and resolution [17].

A recent systematic review highlights the limited validation of IMU-based motion-capture systems in uncontrolled or real-world environments, which continues to hinder their clinical adoption [18]. Additional work involving distributed IMU configurations and virtual biomechanical models indicates that measurement accuracy decreases when fewer sensors are used, and that persistent challenges—such as synchronization, drift, and limited interoperability with established laboratory systems—may restrict translation outside controlled research settings [19,20,21].

Quantifying movement over extended periods is essential for assessing motor dysfunction and designing effective therapeutic interventions. However, kinematic measurements obtained from IMUs can be affected by accelerometer drift, and upper-limb functional tasks often involve complex multi-planar movements across the X, Y, and Z axes, contributing to greater variability in results. By contrast, lower-limb movements generally occur in a single dominant plane, producing more consistent measurements [22]. To mitigate these challenges, many technology companies employ proprietary algorithms that attempt to correct drift and movement-related artifacts. Nonetheless, this creates difficulties for end users, who often cannot independently verify the accuracy or validity of these closed-source methods prior to device adoption [23].

In addition, standardized protocols for sensor placement, calibration procedures, and reporting practices in biomechanical analysis remain limited. Evidence regarding the validity of IMU measurements at the shoulder, elbow, and wrist is inconsistent, particularly when movements involve rotation around the Y-axis [23,24]. Few studies have systematically examined how sensor placement influences the measurement of upper-limb kinematics, resulting in substantial variability due to the anatomical complexity of these joints [25].

Recent estimates from the World Health Organization (WHO) and the Global Burden of Disease (GBD) study indicate that musculoskeletal disorders affect approximately 1.71 billion people worldwide and rank among the top 25 causes of Years Lived with Disability (YLDs). Low back pain is the most significant contributor, accounting for an estimated 70.2 million YLDs globally. These conditions continue to represent a major cause of work absenteeism and long-term disability [26].

Musculoskeletal risks related to posture and movement in the workplace are commonly assessed using standardized observational tools such as the Rapid Upper Limb Assessment (RULA) [27], one of the most widely applied methods for evaluating seated postures. Prior research indicates that fuzzy inference models, when combined with RULA scores, demonstrate high reliability compared with the traditional scoring method, as evidenced by correlation and Bland–Altman analyses [28]. Fuzzy inference systems support the modeling of complex states using interpretable, linguistically based rules, making them well suited for managing imprecise or uncertain information [5]. This capacity to handle subjectivity renders them a valuable complement to observational assessment techniques in movement and ergonomic analysis [28].

In response to these needs, the primary objective of this study is to design, implement, and rigorously evaluate a Bluetooth 5.0-based network of Inertial Measurement Units (IMUs) tailored for upper-limb biomechanical signal acquisition. This work systematically assesses the scalability of the network—including the maximum number of simultaneously connected IMUs, communication range, transmission speed, and sampling-frequency stability—and demonstrates its practical utility through a representative clinical-ergonomic case study. This study contributes evidence that is currently lacking in the literature and directly supports the translation of IMU-based systems into real-world assessment environments.

This manuscript presents the development, integration, and validation of a wireless network comprising six IMU sensors operating under the BLE 5.0 standard. The key contributions of this work are:

- A fully implemented wireless multi-IMU architecture based on asynchronous BLE 5.0 acquisition—addressing a widely acknowledged gap in multi-sensor scalability and synchronization for upper-limb biomechanics.
- A reproducible communication framework, developed in Python (using the Bleak and asyncio libraries) and seamlessly integrated with a user-friendly LabVIEW interface to facilitate real-time visualization, analysis, and system deployment by clinicians and researchers.
- Experimental evaluation of critical performance metrics, including transmission throughput and signal integrity—providing objective benchmarks that are seldom reported in existing commercial or research-grade systems.
- A validated case study demonstrating clinical applicability, where real IMU data were used to develop a fuzzy inference system grounded in the Rapid Upper Limb Assessment (RULA). The resulting model offers interpretable, explainable outputs suitable for physiotherapists and ergonomic risk analysts, enhancing trust and facilitating future adoption.

Collectively, these contributions establish one of the few openly described BLE 5.0 multi-IMU architectures for upper-limb assessment, offering validated performance data and a practical pathway toward scalable, clinically meaningful motion analysis in real-world environments.

## 2. Materials and Methods

### 2.1. Technical Devices

**Figure 1:**
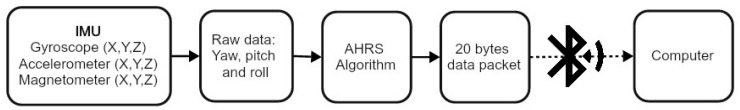
Data flows from IMUs to the computer: sensor measurements, AHRS processing, packet formation, and Bluetooth transmission.

Six Inertial Measurement Units WT901BLECL (WitMotion, Shenzhen, China) equipped with BLE 5.0 were employed, offering a sampling frequency range of 0.1–200 Hz and a battery life of up to 10 h. Each unit integrates an accelerometer, gyroscope, and magnetometer, allowing for the estimation of attitude (the angular deviation between an object axis and the Earth’s horizon) and orientation through Madgwick’s Attitude and Heading Reference System (AHRS) algorithm [29], as illustrated in Figure 1.

**Figure 2:**
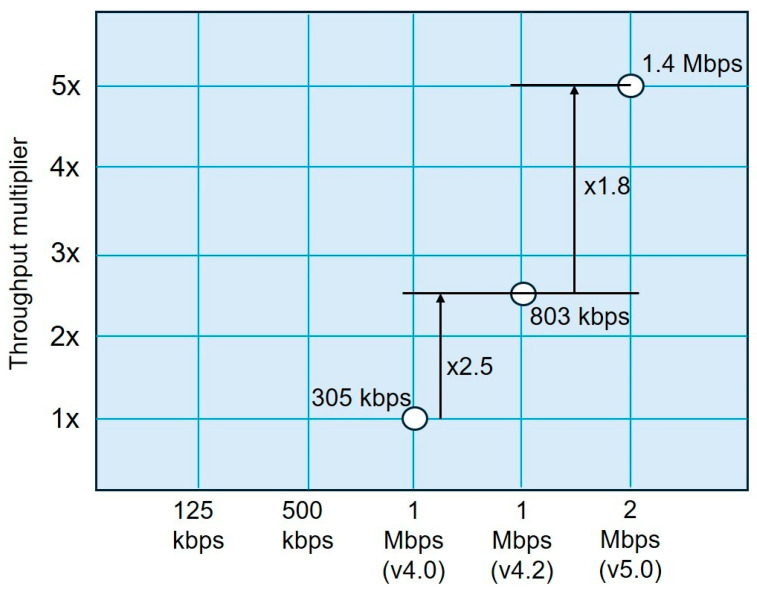
Comparison of BLE throughput across different versions: BLE 4.0 (305 kbps), BLE 4.2 (803 kbps), and BLE 5.0 (1.4 Mbps). BLE 5.0 also provides extended range of modes at 500 kbps and 125 kbps, achieving up to a fourfold coverage increase. Adapted from [31].

The IMU is equipped with the nRF52832 chip (Nordic Semiconductor, Trondheim, Norway), operating over BLE 5.0 and supporting transmission rates of up to 2 Mbps, with a net throughput of roughly 1.4 Mbps (see Figure 2). Extended range modes of 500 kbps and 125 kbps for increased coverage (×4) [30,31].

As shown in Figure 2, Bluetooth 4.2 enabled significant increases in net data throughput, with approximately 700 kbps of this traffic available for practical applications. These improvements were achieved by increasing the maximum packet size, a feature also included in Bluetooth 5.0, which, by doubling the over-the-air transfer rate, achieves a net throughput of around 1.4 Mbps.

### 2.2. Sensor Characteristics and Attributes

The network was integrated with six IMUs, each assigned a name according to its Media Access Control (MAC) address. Table 1 lists the attributes of each sensor, which were mapped in Python within a device dictionary, allowing for easy addition or removal of devices.

The Generic Attribute Profile (GATT) defines a standard for transmitting small data units, known as “attributes,” over a BLE connection. It enables both the retrieval of sensor data and the transmission of commands, returning hexadecimal byte packets containing values such as quaternions, magnetometer readings, temperature, supply voltage, or calibration information, thus broadening the sensor’s potential applications. Each attribute is uniquely identified by a 128-bit Universal Unique Identifier (UUID) [32].

### 2.3. Software Architecture and Communication Framework

Sensor communication was performed in Python 3.10 using the Bleak library (version 0.21.1), which provides cross-platform support and asynchronous communication via the asyncio package (version 0.22.0) [33]. The data acquisition workflow includes:

- Initialization
- Subscription to GATT notifications
- Asynchronous processing of received packets
- Transmission of data via emulated virtual ports to an interface
- Signal monitoring and data storage for post-processing

**Figure 3:**
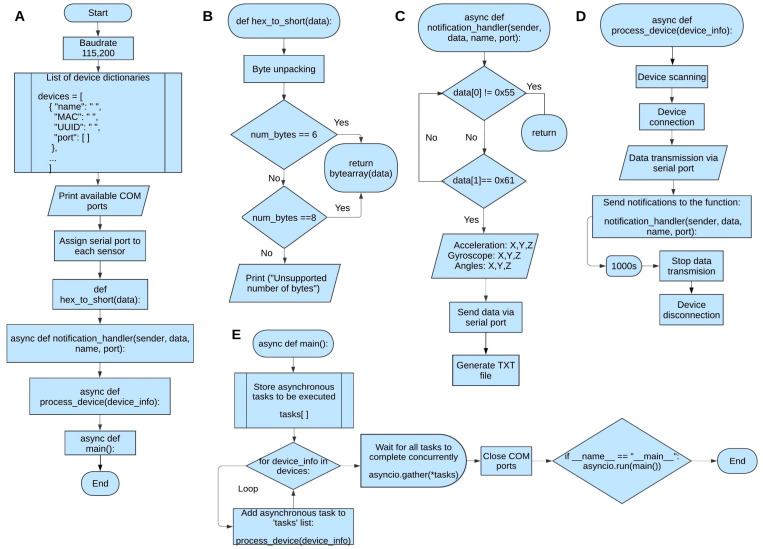
Flowchart of the asynchronous Python script used for multi-IMU BLE communication. (**A**) outlines the programming flow of the main execution; (**B**) presents the flow of the Data Unpacking function; (**C**) describes the Asynchronous Notification Handler, which manages sensor notifications concurrently; (**D**) details the Sensor Connection function executed asynchronously; and (**E**) shows the asynchronous “main” function that orchestrates the overall process.

Figure 3 illustrates the flow diagram of the Python algorithm developed for sensor data acquisition. The algorithm allows simultaneous connections to all sensors while performing asynchronous data acquisition, with the collected data subsequently transmitted through emulated COM ports to the LabVIEW interface.

The components of the flow diagram are as follows:

- Main Execution: This part of the diagram involves importing the necessary libraries, configuring the data transmission rate, and creating a list of device dictionaries for connection. The Communication (COM) ports are initialized and assigned to distinct sensors, ensuring proper execution of subsequent functions.
- Data Unpacking Function: This function is the first to run in the script. It unpacks the data received from the sensors, which arrive as a hexadecimal array. Depending on whether the sensor is transmitting only the variable values—acceleration, angular velocity, and angular position—six or eight bytes may be received. When eight bytes are received, the extra two bytes correspond to quaternion data.
- Asynchronous Notification Handler: This asynchronous function manages sensor notifications concurrently, handling multiple variables from multiple sensors simultaneously. Its input arguments are “sender,” containing the sensor’s MAC address; “data,” containing the unpacked hexadecimal sensor data; “sensor_name,” the name of the sensor; and “port_name,” the number of the port associated with the sensor. Once the data are acquired, the function separates the bytes according to the variables they correspond to, performs the necessary conversions, and obtains the hexadecimal values of the three main variables (acceleration, angular velocity, and angular position). The results are presented in a tabular format on the terminal, concurrently transmitted through the serial port, and also archived in a text file.
- Sensor Connection Function: Like the function described in C, this function must also be handled asynchronously. It receives the argument “device_info,” which contains all the information stored in the sensor list and dictionary. The main objective of this function is to locate the previously mapped sensor(s). Once found, the sensors are defined as clients and connected via an exception handler. Using an anonymous or lambda function, all data extracted from the sensor are sent to the “notification_handler” function until execution is interrupted or the sensor(s) are disconnected.
- Main Function: The “main” function is also executed asynchronously, as it stores in “device_info” the information from each sensor’s “devices” list and sends it concurrently to the “process_device” function. It runs the asyncio event loop until all tasks (*tasks) are completed and closes all ports once execution is finished.

Since BLE cannot guarantee simultaneous transmissions across multiple sensors, the system relies on an asynchronous architecture to preserve temporal coherence. All notifications from the six IMUs are handled within a single event loop, where each data packet is processed and time-stamped using the host’s high-resolution clock. This approach minimizes the impact of BLE latency and enables accurate alignment of samples during post-processing, ensuring stable and coherent multichannel analysis.

**Figure 4:**
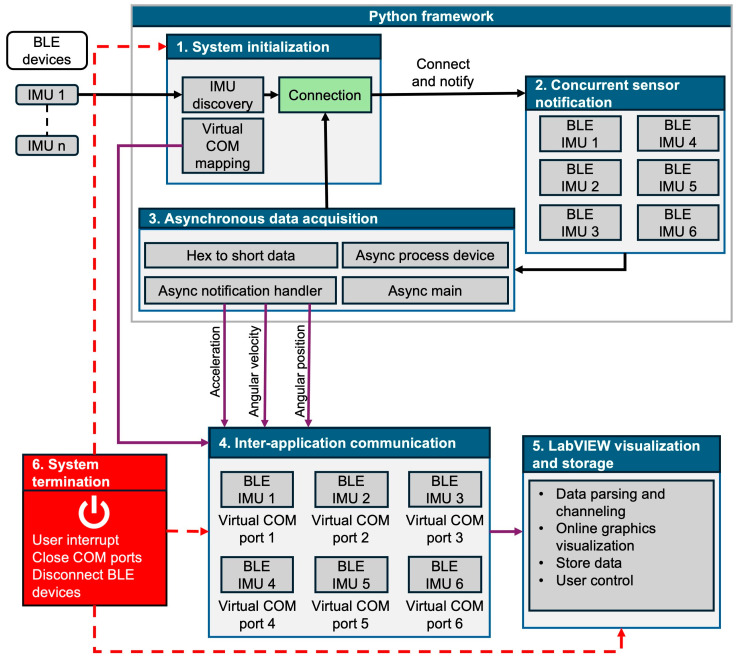
System architecture of the asynchronous multi-sensor acquisition framework. The workflow includes: (1) system initialization; (2) concurrent notifications from six sensors; (3) asynchronous data acquisition; (4) inter-application communication; (5) LabVIEW visualization and data storage; and (6) system termination to close COM ports and disconnect BLE devices.

Figure 4 illustrates the architecture of the multi-sensor data acquisition workflow. Beyond data acquisition, the system was integrated with a LabVIEW interface to enable visualization, management, and storage of the collected data.

**Figure 5:**
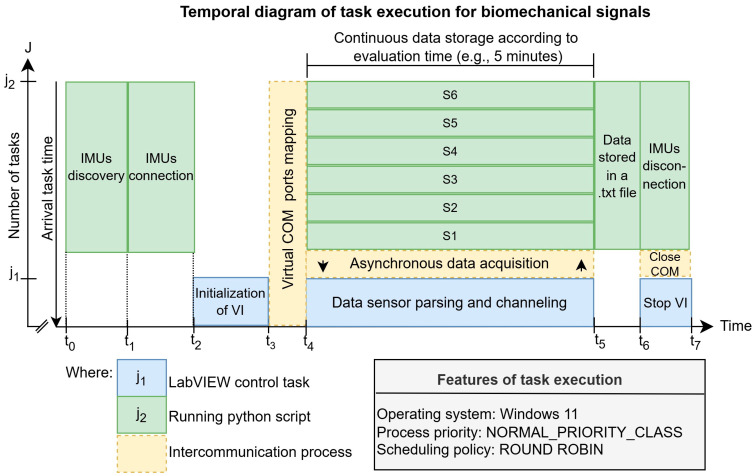
Temporal diagram of task execution for biomechanical signals processing using LabVIEW and Python intercommunication.

An additional perspective on the software architecture is provided by the temporal diagram based on [34], as presented in Figure 5, where $J=\{j_{1},\;j_{2}\}$ represents the set of tasks involved in processing the signals acquired from the six IMUs, and $Times=\left\{t_{0},\;t_{1},\;t_{2},\;t_{3},\;t_{4},\;t_{5},\;t_{6},\;t_{7}\right\}$ represents the set of processing times for these tasks. Control sequencing is managed via LabVIEW, while low-level processing and communication are executed concurrently in a Python script. Communication between these tasks happens through an inter-process mechanism managed by emulated virtual COM ports, ensuring logical synchronization of the data flow.

In the computational context, the temporal diagram begins with the arrival time. This value indicates when the Python task is declared ready and placed in the waiting queue to be executed by the processor. The task starts execution at $t_{0}$, and the time elapsed between $t_{0}$ $t_{1}$ is dedicated to hardware initialization, including searching for the BLE devices previously defined in the script and their MAC addresses. From $t_{1}$ to $t_{2}$, the connection and pairing process is initiated. Once connected, communication with LabVIEW begins $t_{2}$, where the interface allows verification of each sensor’s successful connection. Simultaneously, inter-process communication is established between $t_{4}$. This process maps virtual COM ports to create a bidirectional channel, enabling the transfer of control commands between LabVIEW and Python and ensuring logical coupling of the operating system under a Round Robin scheduling policy with NORMAL_PRIORITY_CLASS.

Starting at $t_{4}$ the continuous acquisition phase. This phase is characterized by the asynchronous acquisition of data from all six sensors. The script concurrently executes two essential subtasks:

1. Asynchronous Acquisition: Reception of data packets from the IMUs.
2. Processing and Routing: Parsing and formatting of the data to ensure a uniform data structure prior to storage.

This cycle continues indefinitely, depending on the evaluator. Experiments of varying durations, for example, five minutes, can be conducted, enabling continuous storage of biomechanical data in txt files, with efficiency dependent on the robustness of the connection.

The termination phase begins $t_{5}$ with the stop of continuous acquisition and closure of the data storage workflow. The LabVIEW task issues the command to stop the Virtual Instrument, triggering the sequential disconnection of the sensors $t_{6}$. Finally, at $t_{7}$ the COM ports used for software communication are closed, marking the complete end of task execution. The clear separation of these stages ensures error traceability and the temporal integrity of the acquired datasets.

**Figure 6:**
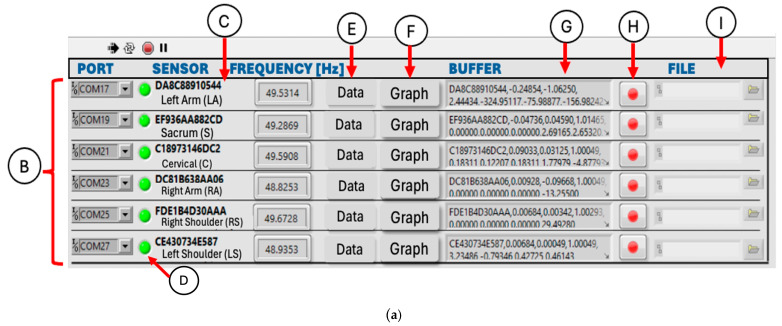

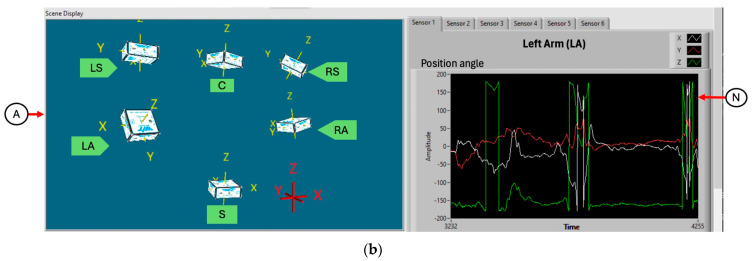
LabVIEW control panel and GUI elements showing: (**a**) information for the six IMU sensors, including port, sensor MAC addresses, frequency, data and graph buttons, buffer, and file-path selector for storing data from the wireless IMU network into a .txt file; and (**b**) 3D visualization of the IMUs along with the corresponding sensor plots.

**Figure 7:**
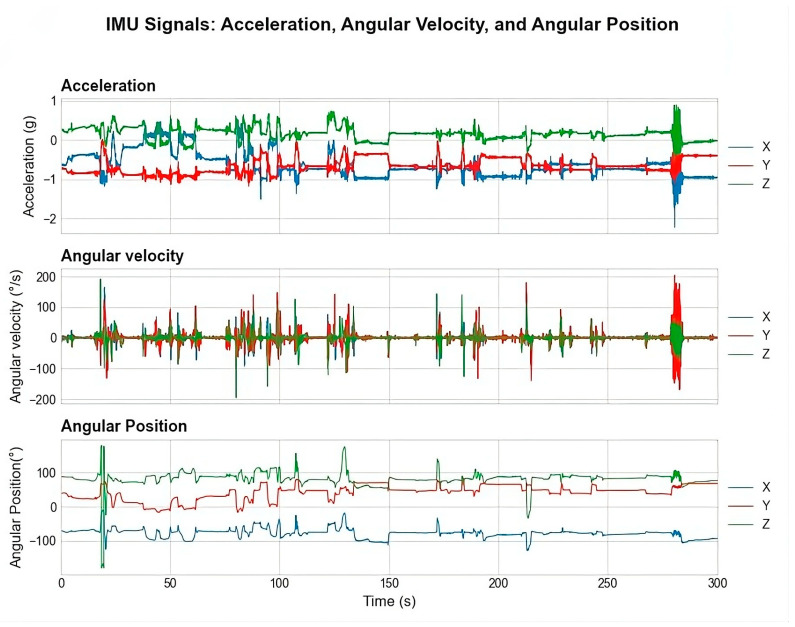
Time-series data stream from the right arm IMU: Three orthogonal orientation variables.

The elements comprising the interface shown in Figure 6a,b are as follows:

- 3D Visualization of the IMUs: The labels indicate the placement of each sensor on the upper limb as follows: LA—Left Arm, LS—Left Shoulder, C—Cervical Vertebra, S—Sacrum, RS—Right Shoulder, RA—Right Arm.
- COM Port: The COM port used by the Python script to receive data from each sensor.
- Sensor Name and Assigned Upper Limb Location
- Connection Status Indicator: Indicates whether the sensor is currently connected.
- Data Button: Opens a window that displays sensor data in tables.
- Graphics Button: Launches a window for online visualization of the acquired signal plots.
- Data Buffer: Shows the incoming data stream from each sensor, enabling monitoring of the received byte packets.
- Data Storage Button: Initiates independent storage of signal data from each sensor in a text file. The file captures the three variables from each IMU, along with their respective components (X, Y, Z), as depicted in Figure 7, and serves as the dataset used for subsequent post-processing.
- File Path Selector: Enables the user to specify the destination .txt file for data storage.
- Average Sampling Frequency: The average sampling rate across all six sensors.
- Upload TXT: Allows loading a previously stored signal for graphical visualization.
- Stop VI: Stops the data acquisition process and intercommunication.
- Start All-Sensors Storage Button: Triggers simultaneous storage of data from all sensors and presents a timer indicating the duration of the ongoing acquisition.
- Online Sensor Plots: Displays real-time angular position signals from all six sensors.

**Figure A1:**
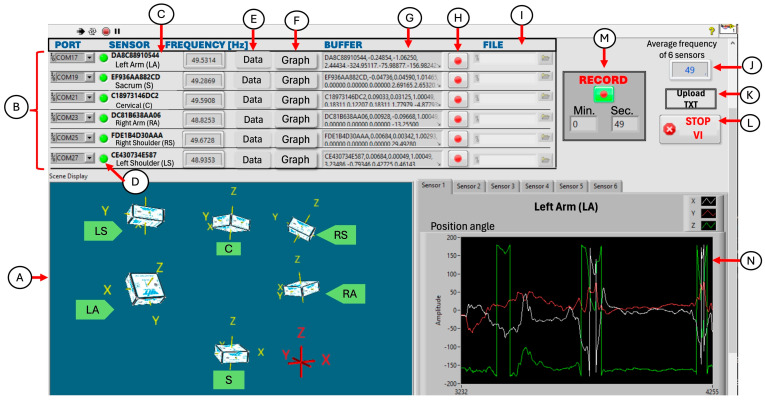
LabVIEW control panel and Graphical User Interface (GUI) for the wireless IMU network (letters A–N) are described in detail in Section 2.3.

In the complete LabVIEW GUI (Graphic User Interface) and control panel shown in Figure A1, the elements corresponding to letters “J, K, L, M” are clearly displayed.

### 2.4. Post-Processing and Case Study

**Figure 8:**
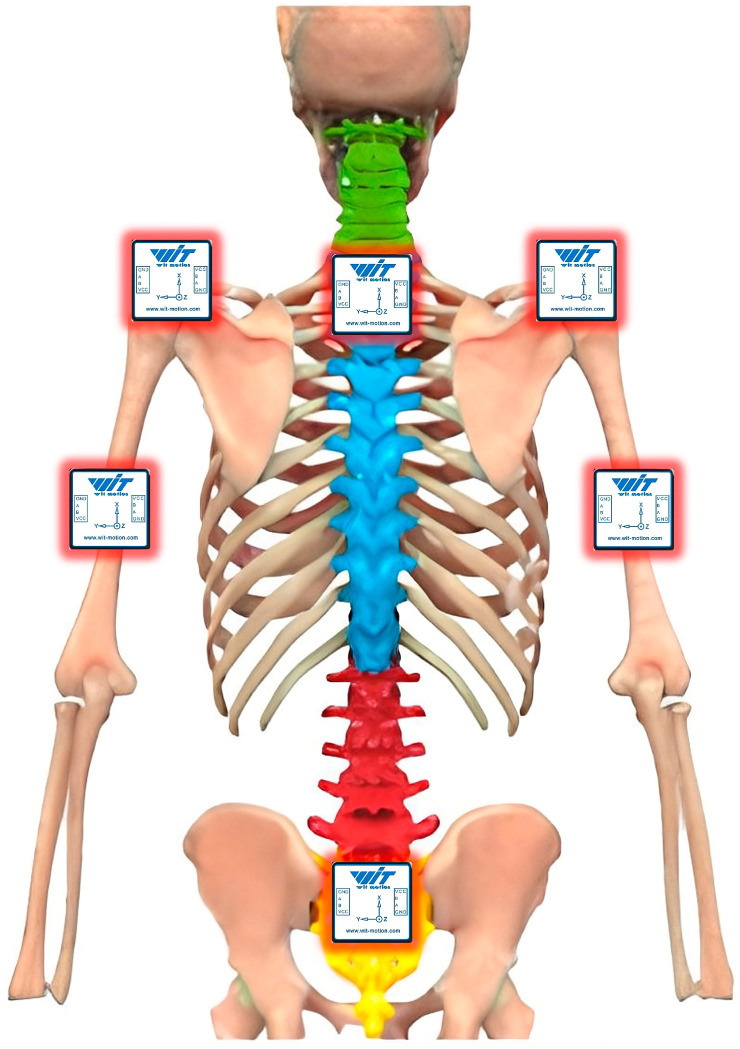
Configuration of the Inertial Measurement Unit (IMU) placement for biomechanical modeling.

Six sensors were positioned at sites near the upper limb, as illustrated in Figure 8. The placement of IMUs is not standardized, but it has demonstrated reliable performance in previous upper-limb evaluations. As described by the authors in [35], IMUs were placed on the left and right arms (LA, RA), left and right shoulders (LS, RS), cervical (C), and sacral (S) regions. These positions were selected to capture the primary kinematic segments involved in upper-limb posture evaluation—arm elevation, abduction, flexion, and rotation—relative to the trunk, consistent with the requirements of the RULA method and previous ergonomic studies.

Furthermore, a comprehensive review of the current literature indicates that certain anatomical regions are the most validated for Inertial Measurement Unit (IMU)-based joint angle estimation [22]. This synthesis, which included 39 validity and 15 reliability studies involving 556 subjects, established that the shoulder and trunk are among the most frequently and successfully investigated regions. These segments consistently exhibit the strongest evidence for both validity (measurement accuracy) and reliability (measurement consistency) in IMU applications.

Moreover, some authors have noted that no studies have specifically investigated the importance of IMU sensor placement in upper-limb motion measurements [25]. Nevertheless, recommended sensor locations include the dorsal side at the distal end of the forearm, the upper arm, the lateral aspect of the upper arm, the scapula (cranially, along the spine), thorax, and trunk [23,25,36].

**Figure 9:**
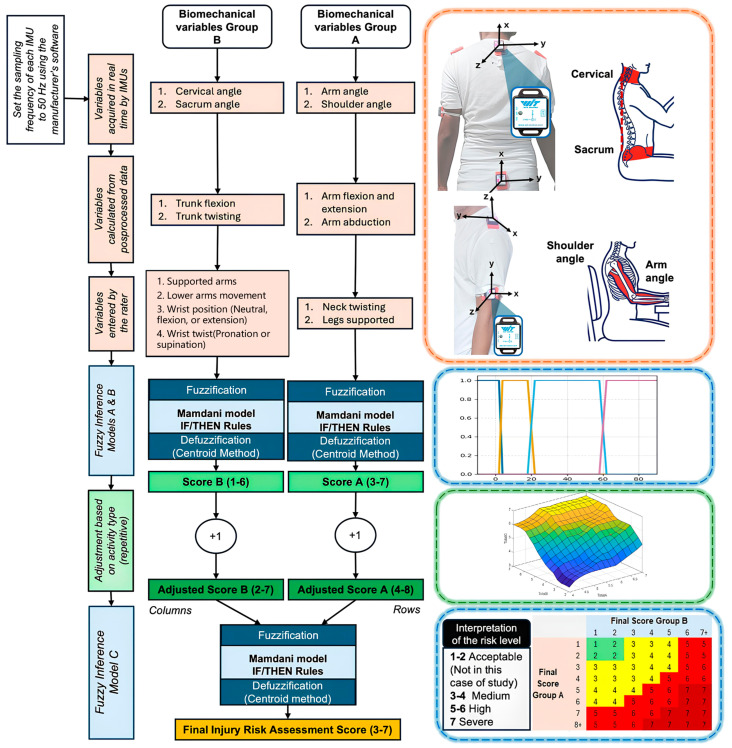
Overview of the three-stage fuzzy logic model for RULA-based biomechanical risk scoring. The first stage integrates IMU-acquired variables and evaluator inputs. The second stage fuzzifies these variables using the Mamdani approach and applies a score adjustment for repetitive activity. The third stage fuzzifies the resulting scores for Group A and Group B to produce the final injury-risk assessment.

Figure 9 presents the overall system diagram for evaluating the sensor network based on upper-limb movements, from setting a sampling frequency of 50 Hz to assessing the injury risk level using the RULA method adapted to fuzzy inference models.

Studies have shown that human upper limb involuntary movements, especially during postural tasks, predominantly contain frequency components between 10 and 15 Hz [37]. A sampling frequency of 50 Hz is sufficient to accurately capture these movements without aliasing, as previous studies in biomechanics have successfully used wireless IMU sensors at similar sampling rates, confirming their adequacy for ergonomic assessments, and this is sufficient for Human Activity Recognition (HAR) tasks [4,6,7,23,38].

Moreover, sampling frequencies between 25 and 50 Hz have been recommended for daily activity monitoring, providing an adequate balance between measurement accuracy and data transmission or power consumption in wearable IMU-based systems. Using a higher frequency than required may unnecessarily increase computational load without improving the quality of the results [39,40]. In addition, studies have validated the reliability of inertial sensors for biomechanical and ergonomic applications—both spatiotemporal and kinematic—using IMU—based gold standard systems such as MatScan by Tekscan, Optotrak 3020, Free4Act by Vicon Motion Systems, XSens, and Axivity AX3 by GAITRite, which operate within the 25–50 Hz range [41,42].

The procedure for biomechanical evaluation was conducted as follows:

1. Before sensor placement, calibration was performed using the manufacturer’s software, allowing sampling frequencies to be set from 0.1 Hz to 200 Hz.
2. Sensors were placed at their corresponding locations using a shirt, which includes adaptations for securing the IMUs.
3. Each IMU was mapped to its designated emulated virtual port.
4. The LabVIEW interface was launched, enabling data from each sensor to be received via the Python script.
5. The connectivity and data transmission of each sensor were confirmed, and it was verified that the sensors operated at the designated sampling frequency.
6. Sensor signals were saved in text files, capturing acceleration (X, Y, Z), angular velocity (X, Y, Z), and positional angle (X, Y, Z) data.
7. During post-processing, datasets were aligned to ensure uniform length (15,000 data points), followed by component analysis to compute the movement angles of the upper-limb segments—arm flexion, extension, and abduction—along with trunk tilt and rotation.
8. The acquired data were assessed using a Fuzzy Inference System implemented in Simulink.
9. Injury risk levels were evaluated using the RULA method.
10. The evaluator provided recommendations based on expert knowledge.

### 2.5. Biomechanical Component Analysis

**Figure 10:**
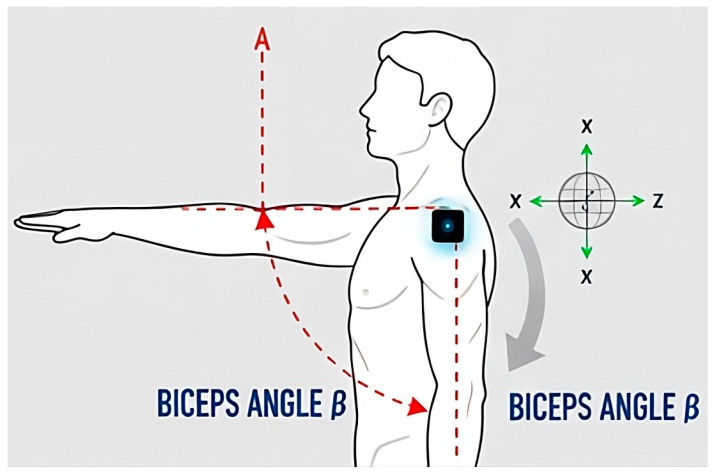
Components of arm flexion and extension.

An adjustment must be made between the angle measured by the arm sensor and that measured by the shoulder sensor. This is necessary because if only the readings from the arm sensor were considered, it would not discriminate between a tilted trunk and a trunk positioned off the vertical axis (see Figure 10).

To synchronize the datasets and ensure uniform data length across all files, interpolation was used to extend datasets with fewer samples to 15,000 data points. Conversely, datasets surpassing this sample size were truncated to conform to the specified limit. This procedure facilitated consistency among all signals. Consequently, when computing the resultant flexion angle utilizing shoulder and arm sensor data, a single file comprising 15,000 synchronized samples was generated for both flexion/extension and abduction movements. The aligned dataset was subsequently utilized for inference modeling.

**Figure 11:**
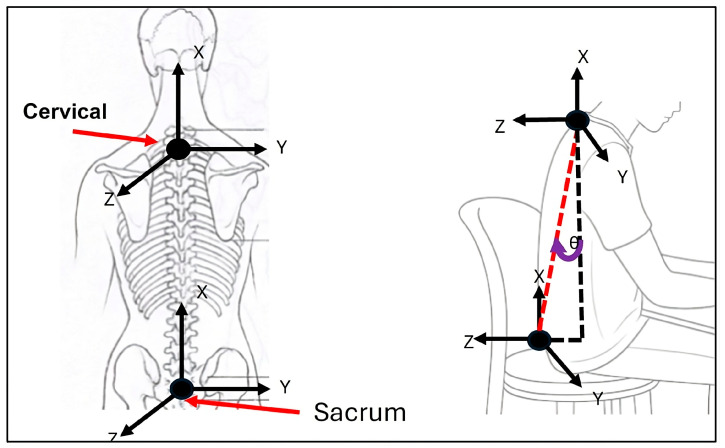
Trunk inclination/twisting.

The data on the trunk inclination angle and its position require a component analysis, as illustrated in Figure 11. This figure demonstrates how the relative placement of the sensors facilitates the estimation of trunk inclination. As with the calculation of arm flexion and extension, it is essential to determine whether the trunk is tilted, centered, or rotated. Consequently, by adding or subtracting the components along the X and Y axes, the resulting inclination angle can be determined. Additionally, as in the case of group A, data alignment must also be performed for group B, utilizing measurements obtained from sensors located on the sacrum and cervical regions.

### 2.6. Fuzzy Inference Models

The fuzzy inference model implemented in this study is of the Mamdani type and comprises four stages: (1) fuzzification of the input variables using membership functions, (2) rule evaluation, (3) aggregation of the rule outputs, and (4) defuzzification [43].

The most commonly employed membership functions include triangular, trapezoidal, singleton, sigmoid, and Gaussian types. In accordance with the contextual and semantic knowledge available, these membership functions can be directly adjusted based on expert judgment and intuitive reasoning; this procedure is applied in the present study [44,45].

**Figure A2:**
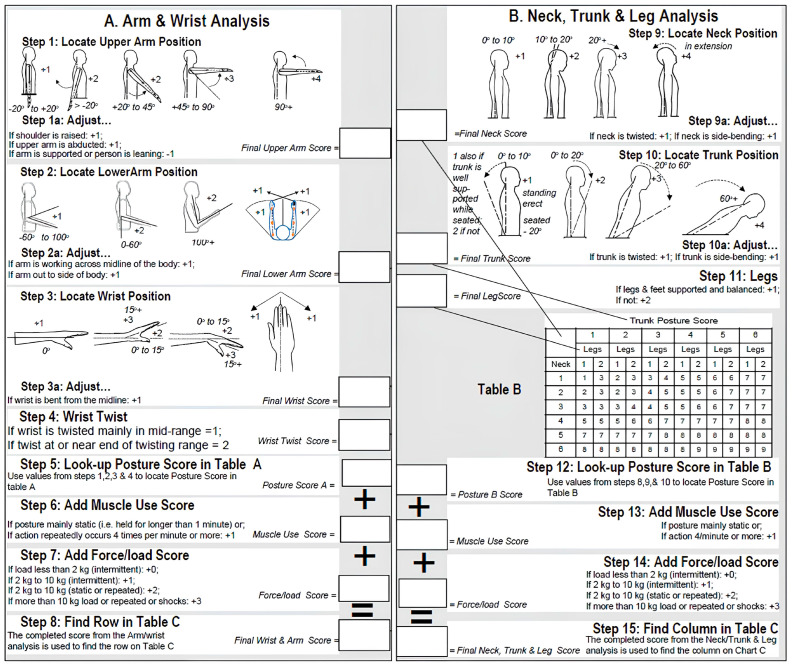
Groups A and B according to RULA method. Adapted from [46].

To adapt the RULA method for use within fuzzy inference models, the upper limb is partitioned into two groups: Group A, which includes the shoulders, arms, forearms, and wrists; and Group B, which encompasses the trunk, legs, and neck (Figure A2). A third fuzzy model is subsequently used to determine the final risk level assessment based on the outputs of Groups A and B [46].

**Figure 12:**
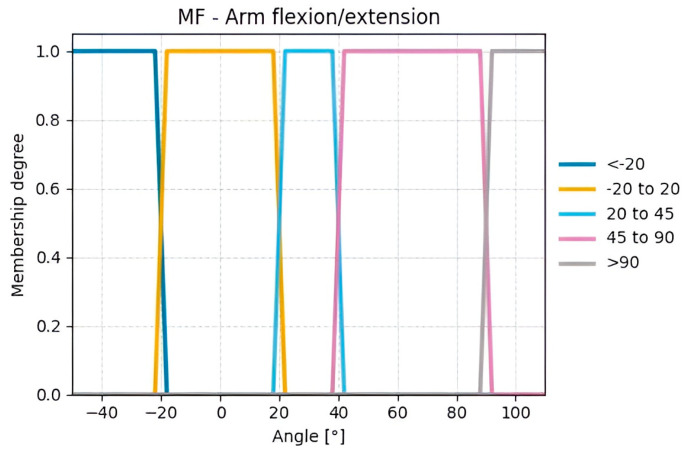
Membership functions for the calculated Flexion/Extension angles.

**Figure 13:**
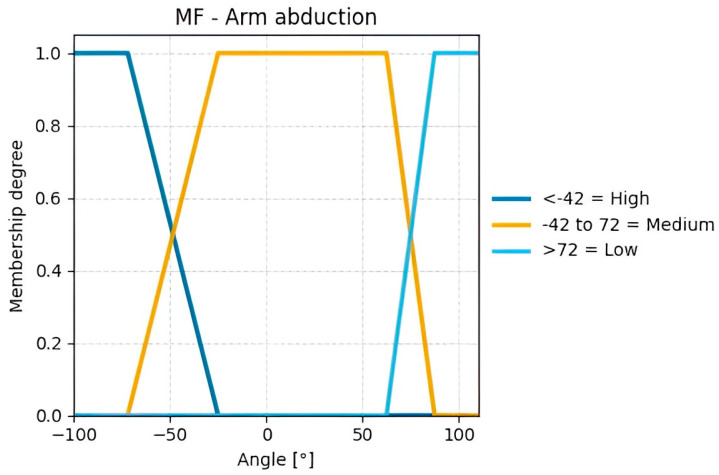
Membership functions for the calculated Arm Abduction angles.

From the calculated variables illustrated in Figure 10, the input variables were defined. For Group A, the input variables are arm flexion/extension (Figure 12) and arm abduction (Figure 13).

**Figure A3:**
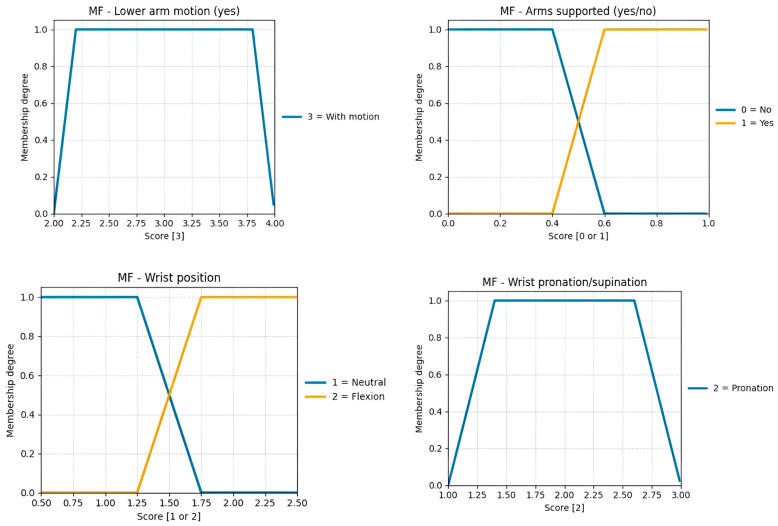
Input membership functions of the fuzzy inference models used for RULA-based upper limb risk assessment entered by the rater for group A.

**Figure A4:**
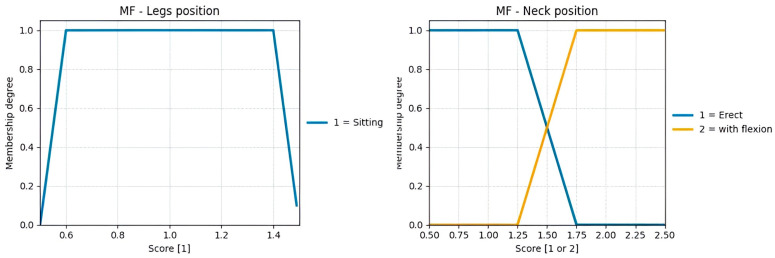
Input membership functions of the fuzzy inference models used for RULA-based upper limb risk assessment entered by the rater for group B.

The variables of Groups A and B were fuzzified using the centroid method due to its wide acceptance, ease of implementation, and reliable results. Trapezoidal membership functions were employed because of the nature of the case study, as human movements are not gradual or slow but abrupt and rapid, resulting in well-defined plateaus for each value range, unlike triangular or Gaussian functions (See Figure A3 and Figure A4 with the remaining membership functions from variables entered by the rater).

As previously indicated, the rule base was constructed using a Mamdani-type fuzzy inference structure [43], drawing on expert knowledge and a rigorous examination of the angular movements specified by the RULA evaluation procedure. For this purpose, Table A1 has been included to present the complete set of rules used in the fuzzy inference models.

**Figure 14:**
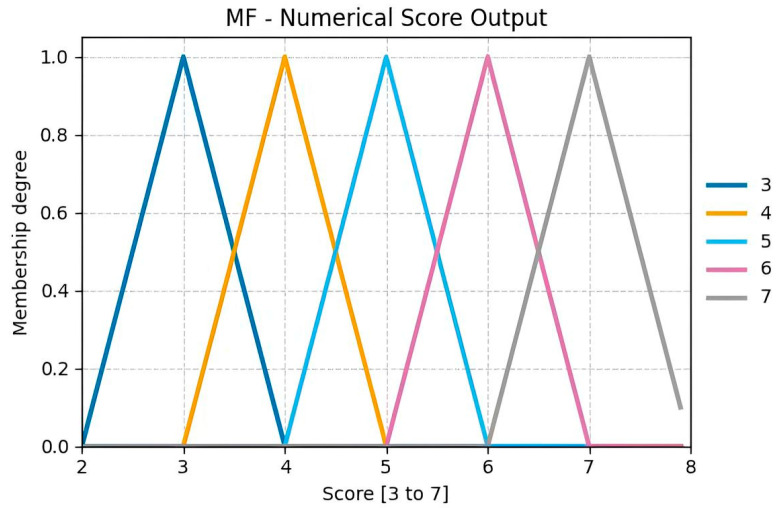
Numerical score output for Group A.

The triangular membership functions (MFs) used for the outputs in Figure 14 (ranging from 3 to 7) correspond to the scores defined in Figure A2 of the RULA methodology applied to this case study [46]. These functions cover narrow intervals, providing a smooth and logical transition between consecutive numerical values. This design maintains low computational complexity while clearly distinguishing each category.

**Figure 15:**
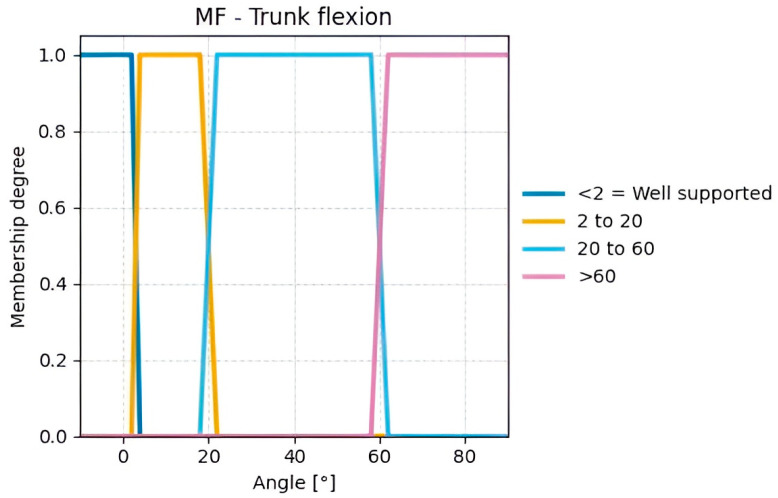
Membership functions for the calculated Trunk flexion angles.

**Figure 16:**
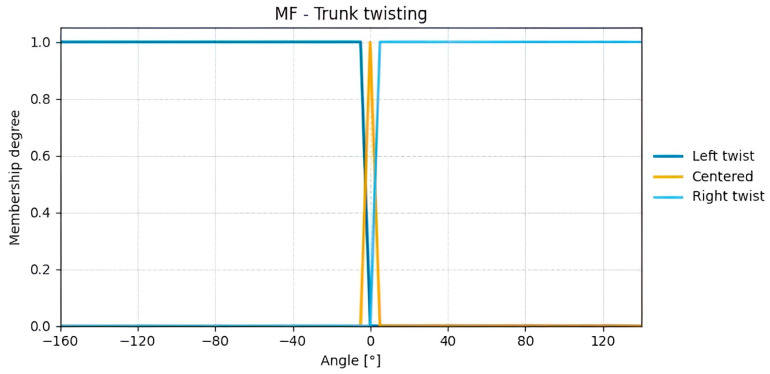
Membership functions for the calculated Trunk twisting angles.

For Group B, the input variables calculated from processed data are trunk flexion (Figure 15) and trunk twisting (Figure 16). Given that a proper posture naturally involves keeping the trunk centered, a tolerance range of ±5° around 0° is considered acceptable. When this angle is exceeded, the subject is twisting the trunk to the right or left.

**Figure 17:**
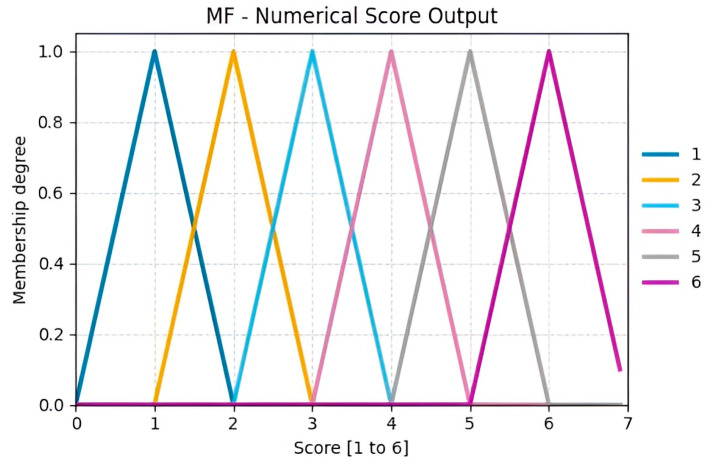
Output Score Group B.

The triangular MFs used for the outputs in Figure 17 (ranging from 1 to 6) correspond to the scores defined in Table B contained within Figure A2 of the RULA methodology applied to this case study [46]. These functions cover narrow intervals, providing a smooth and logical transition between consecutive numerical values. This design maintains low computational complexity while clearly distinguishing each category.

**Figure A5:**
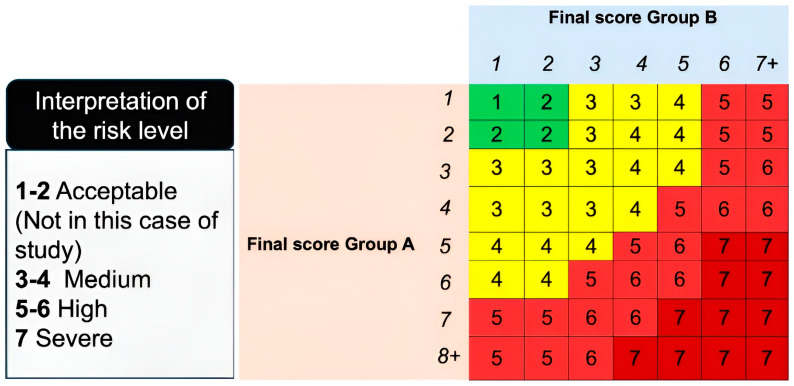
Injury risk level interpretation for the final evaluation of Groups A and B.

Step 6 of the RULA method, illustrated in Figure A2, states that if a static posture is maintained for more than one minute, one additional point should be added to the final score for both Group A and Group B. For Group B, this adjustment is explicitly mentioned again in Step 13. Consequently, the new score ranges become 4–8 for Group A and 2–7 for Group B. According to Figure A5 of the RULA method, the final risk assessment for prolonged sitting postures ranges from medium (3–4) to severe (7).

**Figure 18:**
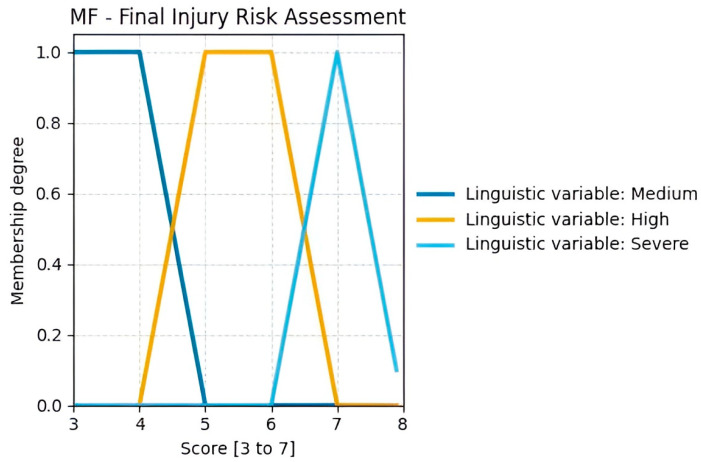
Total RULA outcomes used to assess the final injury risk level.

The third fuzzy inference model, used to obtain the final risk evaluation, takes as inputs the scores from Groups A and B, each increased by 1, to account for the evaluation of a static task. Two trapezoidal membership functions were used: one covering values 3 and 4, corresponding to a medium risk level, and another covering values 5 and 6, corresponding to a high-risk level. A triangular membership function was employed for the severe risk level, which corresponds exclusively to the value 7 (See Figure 18).

**Figure 19:**
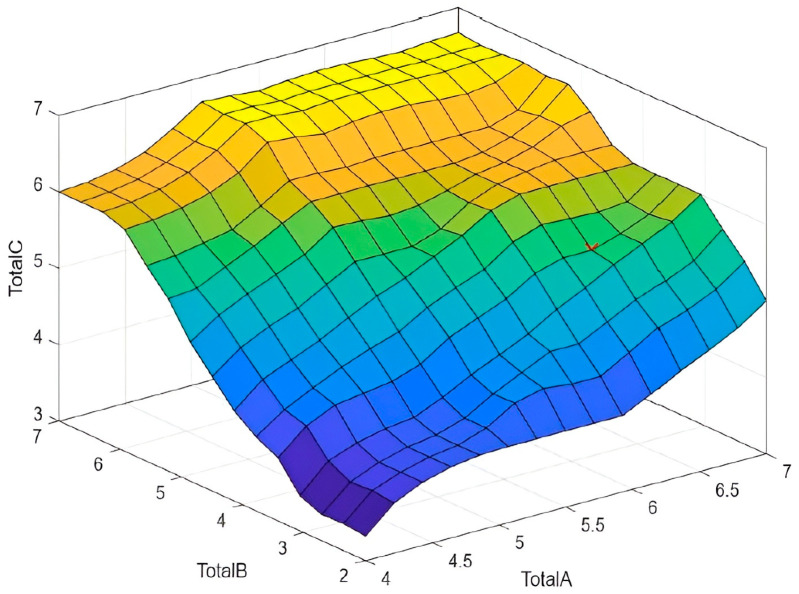
Fuzzy control surface for final injury risk assessment (Total C) as a function of adjustment posture scores (Total A and Total B) based on RULA for sitting tasks.

Figure 19 shows the control surface of the third fuzzy inference model, corresponding to the final matrix logic of the RULA method. This three-dimensional surface maps the nonlinear relationship between the two adjusted input variables.

The surface shows a clear tendency for the final risk to increase as both inputs (Total A and Total B) increase, consistent with RULA’s ergonomic logic. The output range (Total C) spans from a minimum of 3 (for low values of A and B) to a maximum of 7, indicating the transition from medium to severe risk. The smoothness and curvature of the surface are inherent to fuzzy logic (due to the interpolation of membership functions and Mamdani rules), providing a finer, continuous risk gradation compared to the discrete stepwise mapping of the original RULA table.

The control surface demonstrates the effectiveness of defuzzification (centroid method) in converting the aggregated output of the IF/THEN rules into a crisp and continuous score. The shape of this surface confirms that the inference engine reproduces the logic of RULA’s Figure A5 while providing enhanced risk resolution and allowing smooth interpolation between risk thresholds, which is advantageous for online monitoring systems based on inertial sensor data.

## 3. Results

### 3.1. Technical Evaluation of the Network

The system evaluation was based on:

- Comparison between BLE 4.0 and BLE 5.0 specifications.
- Measurement of range in indoor and outdoor environments.
- Analysis of the Received Signal Strength Indicator (RSSI) at different distances.
- Estimation of transmission speed with varying numbers of sensors and sampling frequencies.
- Determination of the maximum number of sensors that can be connected simultaneously.

#### 3.1.1. Range

The inclusion of a comparison between BLE 4.0 and BLE 5.0 specifications, shown in Table 2, serves to justify the deliberate selection of BLE 5.0 as the foundation for the proposed communication network. Although contemporary commercial IMU systems typically offer BLE 5.0 compatibility, these platforms often rely on proprietary firmware which restricts flexible, scalable integration of multiple sensors. In contrast, the solution presented in this work capitalizes on BLE 5.0’s increased data bandwidth (2 Mbps), minimized connection intervals, and advanced concurrency capabilities, enabling robust asynchronous acquisition and logical synchronization of six IMUs. This level of open, multi-sensor interoperability with real-time performance is not inherently available in current commercial options, thereby underscoring the technical significance and application potential of our developed framework.

**Figure 20:**
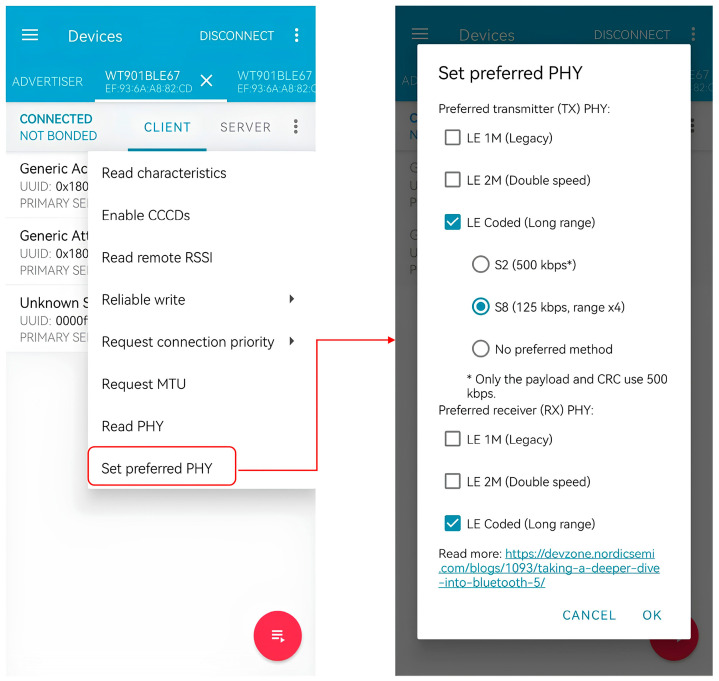
Screenshot of the *nRF Connect* application (version 4.29.1) displaying the PHY settings, where the radio physical layer is configured to the Long Range (LE Coded) mode.

Range evaluation confirmed that the BLE 5.0 network maintained stable communication at distances of up to 105 m in outdoor conditions and approximately 40 m in indoor environments with structural obstacles, notably exceeding the ranges typically reported for BLE 4.0. Measurements were obtained using the *nRF Connect* mobile application, developed by the manufacturer of the IMU’s integrated chipset, as shown in Figure 20. The long-range configuration was established by manually setting the link to PHY Long Range (Coded PHY, S = 8). During testing, the connection parameters displayed in *nRF Connect* verified that the sensor node and the central device successfully established and maintained the Coded PHY link, confirming that long-range mode was correctly enabled on the selected SoC and firmware.

**Figure 21:**
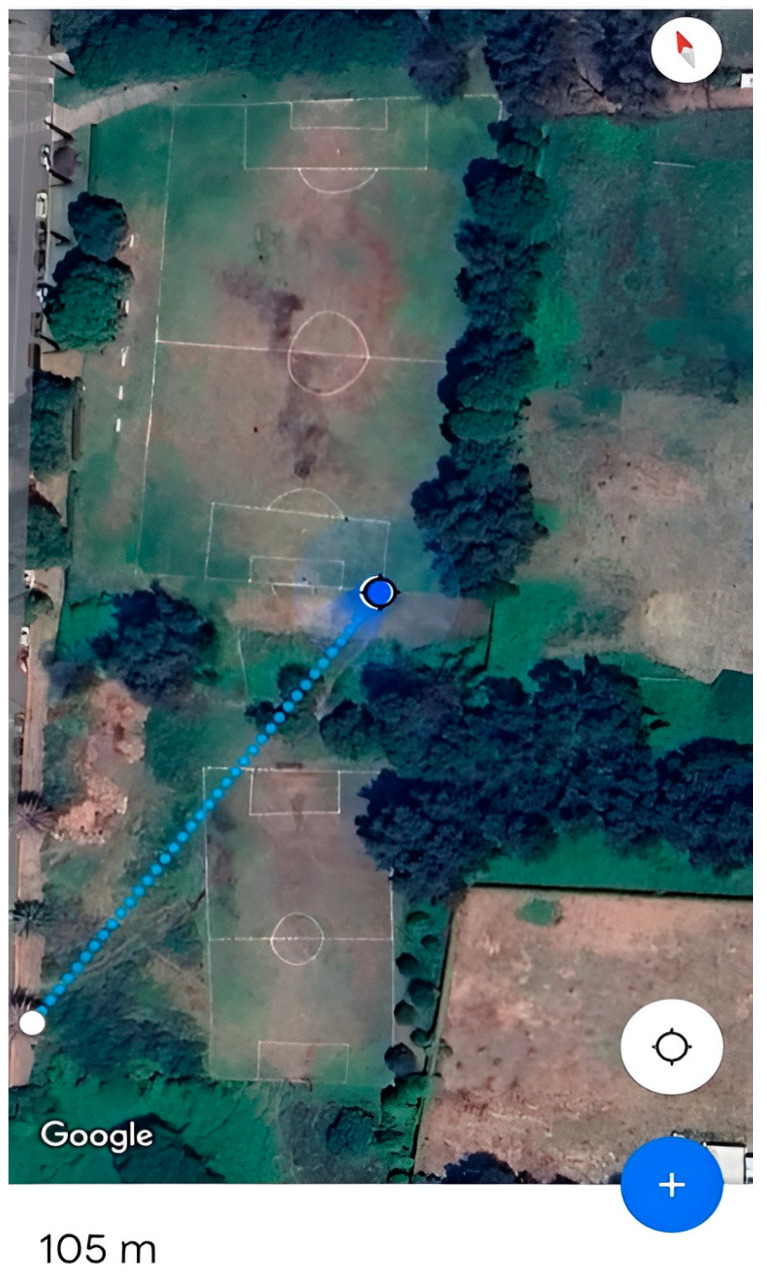
Maximum Line-of-Sight (LOS) range performance of the IMU wireless communication link (Outdoor test).

**Figure 22:**
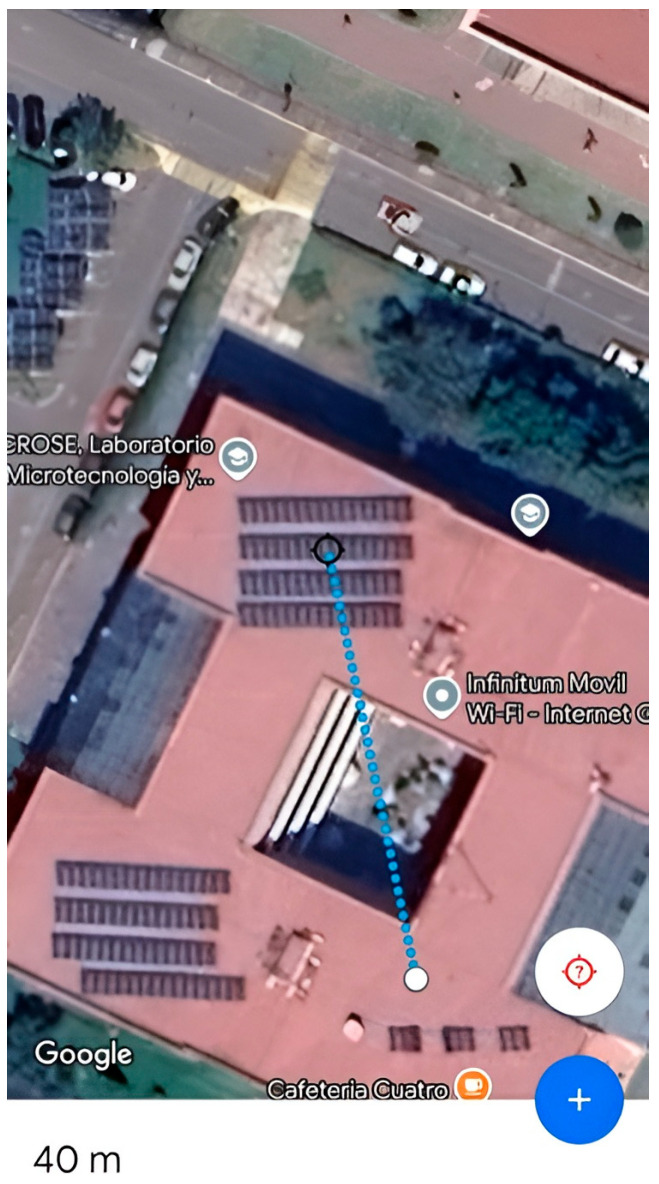
Maximum Line-of-Sight (LOS) range performance of the IMU wireless communication link (Indoor test).

Figure 21 illustrates the area in which the IMU connection range was measured, achieving a maximum distance of approximately 105 m using the 125 kbps extended transmission mode. Indoors with obstacles, a stable connection was maintained up to 40 m (see Figure 22).

#### 3.1.2. Transmission Speed and Received Signal Strength

The sensors were configured using the manufacturer’s software at a sampling frequency of 50 Hz. Each data packet consists of a 20-byte sequence, and Equations (1) and (2) allow the calculation of the transmission speed.

(50 packets/s) × 20 (bytes/packet) = 1000 bytes/s(1)

(1000 bytes/s) × 8 (bits/byte) × 6 IMUs = 48 kbps(2)

**Figure 23:**
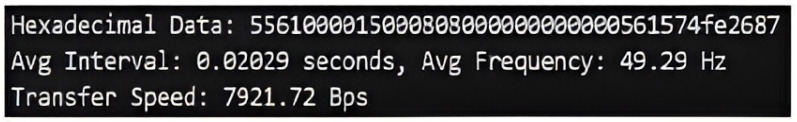
Measurement of data transmission rate (Throughput) for a single IMU.

An evaluation of transmission speed was conducted using a Python script, which showed that at approximately 30 m with obstacles, each IMU achieved a speed of approximately 8 kbps, as illustrated in Figure 23. Table 3 shows theoretical values vs. measured BLE throughput per IMU and aggregate (LOS/NLOS, near/far).

**Figure 24:**
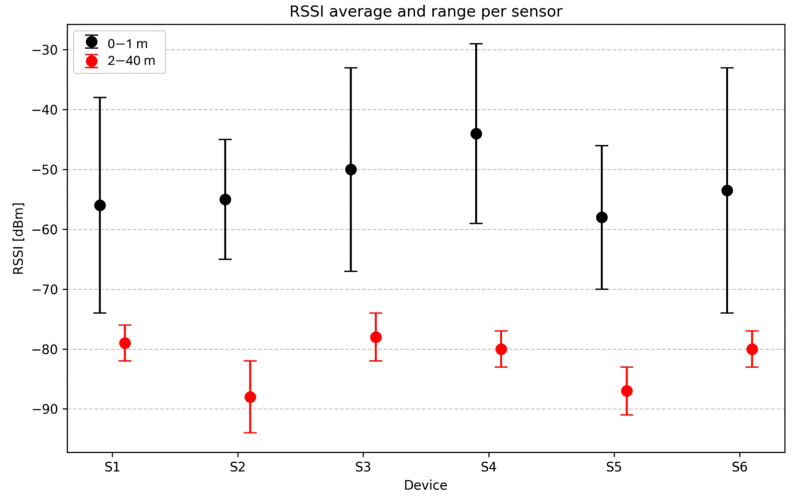
Distribution of the Received Signal Strength Indicator (RSSI) for six BLE IMUs at two distance ranges: short range (0–1 m) and long range (2–40 m). The values represent the signal’s variation range in a close environment; the wide dispersion of RSSI values can be observed even at short distances.

The Received Signal Strength Indicator (RSSI) was measured using the same Nordic Semiconductor application in an environment with obstacles. The results for each sensor are shown in Table 4. When the sensor was positioned no more than one meter from the receiving device—a MSI GF63 THIN laptop with a Bluetooth 5.2 adapter—the RSSI ranged from −29 dBm to −74 dBm. This wide variation, observed in Figure 24, is typical for commercial sensors from the same manufacturer, yet the signal remains strong to very strong, despite ideal RSSI values being between −30 dBm and −55 dBm [47].

The RSSI results showed variability among sensors, with values ranging from −29 dBm to −74 dBm at distances under one meter, reflecting the heterogeneity among commercial devices.

#### 3.1.3. Comparative Statistical Evaluation of Sensor Performance

To evaluate the consistency and stability of data transmitted by the six inertial measurement units (IMUs), a statistical analysis was conducted using descriptive and inferential metrics. Each sensor was tested under identical conditions for 5 min at a sampling frequency of 50 Hz, resulting in an expected 15,000 samples per measurement. The tests were carried out indoors, within an academic building characterized by concrete walls with wood cladding, tiled floors, and glass doors. Measurements were performed under line-of-sight (LOS) conditions, with the devices diagonally across the room, while two Wi-Fi access points operating in the 2.4 GHz band remained active.

The objective of this analysis was to identify potential deviations in data transmission among sensors and determine whether all devices exhibited comparable performance under the same environmental and network conditions. Descriptive statistics—including the arithmetic mean, standard deviation, variance, and coefficient of variation, among others—were calculated for each sensor to quantify variability and dispersion (See Table 5). The results show that sensors 3, 4, 5, and 6 have higher means (15,286.8–15,556.3) and lower variability than sensors 1 and 2, which exhibit larger standard deviations (1209.6 and 1297.15, respectively). These findings suggest that response stability and calibration consistency are higher in Sensors 3–6, whereas Sensors 1 and 2 show greater dispersion and potential bias relative to the expected value (15,000).

Additionally, a non-parametric Kruskal–Wallis test [48], followed by Dunn–Bonferroni post hoc comparisons [49], was applied to determine whether significant differences existed among the distributions of the six sensors.

The results revealed a significant effect of the sensor factor on the measured values (H = 42.12, *p* < 0.001). This indicates that at least one of the sensor distributions differs from the others.

To identify which pairs of sensors showed significant differences, a post hoc analysis using Dunn’s test with Bonferroni correction was conducted. The pairwise comparison results (Table 6) showed that the most significant differences were found between Sensor 1 and Sensor 3 (*p* = 0.0046), Sensor 1 and Sensor 6 (*p* = 0.00025), Sensor 2 and Sensor 3 (*p* = 0.0000081), Sensor 2 and Sensor 4 (*p* = 0.00123), Sensor 2 and Sensor 5 (*p* = 0.00284), and Sensor 2 and Sensor 6 (*p* = 0.0000002).

No statistically significant differences were observed among Sensors 3, 4, 5, and 6 (*p* > 0.05), suggesting that these sensors have similar median responses.

Sensor 6 is the most stable and consistent, exhibiting very low data dispersion. Sensor 3 is also highly stable and performs well. Sensor 4 shows stability with only slight variation. Sensor 5 displays moderate variability, which remains acceptable. In contrast, Sensors 1 and 2 show notable dispersion.

While the literature provides limited guidance on an explicit coefficient of variation (CV) threshold for BLE-based multi-IMU systems, studies of wearable inertial measurement units under realistic conditions have reported acceptable variability levels of around 10%. For instance, the authors of [50] assessed the between-day reliability of HIKOB FOX IMUs in spinal motion analysis and found an average CV of approximately 10% for dynamic stability and trunk movement variability tasks, even under complex motion conditions.

In this study, our maximum observed CV (8.14%) remains below this empirical 10% limit, suggesting that the recorded variability is within acceptable bounds for biomechanical applications. Furthermore, this level of variation is consistent with prior work reporting reliable IMU performance in BLE-based acquisition setups [51,52], where communication factors, such as throughput and packet loss, did not significantly compromise signal integrity. Therefore, the obtained CV values support the stability and consistency of the proposed BLE multi-sensor network for biomechanical monitoring.

#### 3.1.4. Data Loss and Sensor Network Efficiency

**Figure 25:**
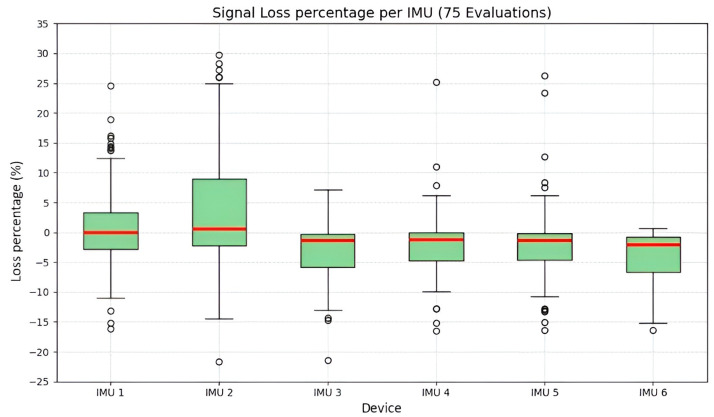
Boxplot of acquisition loss (%) per IMU. Red lines show medians, and outliers are marked by small circles. Positive values indicate true signal loss.

Figure 25 presents a box-and-whisker plot of the distribution of data loss percentages—or residual count error—across the six IMUs, with 75 evaluations per sensor, during the acquisition of upper-limb biomechanical signals across three postural experiments involving 25 participants. This metric quantifies the difference between the number of data packets received and the expected total of 15,000 samples for a 5 min acquisition period at a sampling frequency of 50 Hz. It is important to note that negative values below 0% result from the acquisition methodology, indicating an excess of collected data due to temporal variance associated with manual termination of data processing and asynchronous sensor disconnection. Conversely, positive values represent effective signal loss within the acquisition time window.

The median (Q2) for all IMU clusters is around 0%, ranging from 0.56% to −2.05%, as shown in Table 7. Although the mean is skewed by outliers due to data excess—mainly observed in IMUs 1, 2, 4, and 5—the values remain within ±5%, which is considered acceptable for BLE transmission.

The Interquartile Range (IQR) is the primary indicator of transmission stability. IMU 2 shows the greatest dispersion, with an IQR of 11.25%, indicating the most volatile behavior and greater susceptibility to fluctuations from asynchronous termination or data loss. In contrast, IMU 5 exhibits the narrowest IQR of 4.40%, identifying it as the most stable sensor in terms of transmission and disconnection control. Its measurements fluctuate within ±2.20% around the median, meaning that data loss remained relatively stable, with only slight variations observed in some evaluations.

The analysis of outliers is critical, as they represent isolated transmission failures. Although the network demonstrates overall robustness, IMUs 2 and 5 require firmware or protocol optimization to reduce the likelihood of data loss in noisy or high-interference environments.

**Figure 26:**
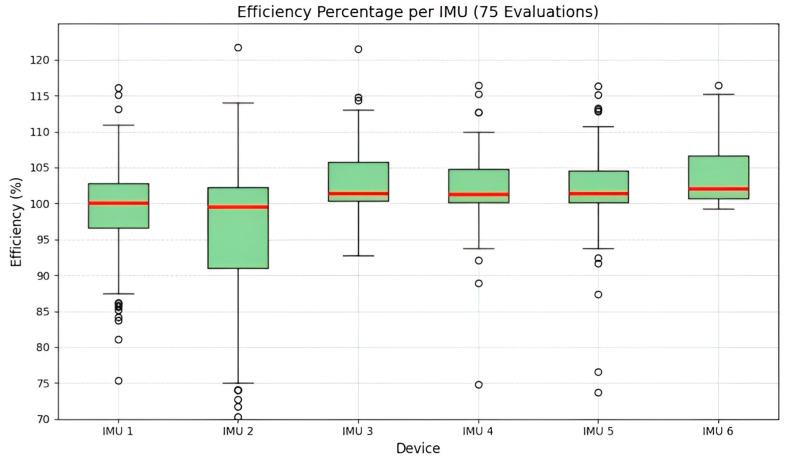
Boxplot of data acquisition efficiency percentage per IMU. Red lines show medians, and outliers are marked by small circles. Most IMUs exhibit a median efficiency close to 100% of the expected data.

Figure 26 presents a box plot of data-acquisition efficiency for the six IMUs across 75 evaluations. Efficiency is defined as the ratio of successfully acquired data points to the total expected samples (15,000). Most IMUs exhibit a median efficiency close to 100% of the expected data. Specifically, IMUs 1, 3, 4, 5, and 6 show median values slightly above 100%, a positive deviation attributed to potential temporal variance induced by the operator (delay in stopping the acquisition), resulting in an excess number of samples beyond the expected count. The narrow interquartile ranges observed in most devices, as reported in Table 8, indicate low dispersion and high consistency in data performance throughout most trials.

The efficiency profile confirms that the sensor network operates with a highly reliable acquisition rate, achieving an average data capture rate of 99–103.71% relative to the expected sample count. The only exception is the outlier values observed in IMU 2, which minimally compromise the sampling frequency required for biomechanical analysis. The slight positive bias is considered acceptable, as it represents redundant rather than missing data, and can be effectively mitigated through data alignment during post-processing.

### 3.2. Biomechanical Evaluation of the Upper Limb in 25 Participants

As part of the sensor network evaluation, using the integrated system based on the RULA method for upper limb assessment, trial conditions were implemented to ensure consistency and reliability of the measurements.

#### 3.2.1. Trial Conditions

To ensure data integrity throughout the process, inclusion and exclusion criteria were applied to every recorded sample, as detailed in Table 9.

**Figure 27:**
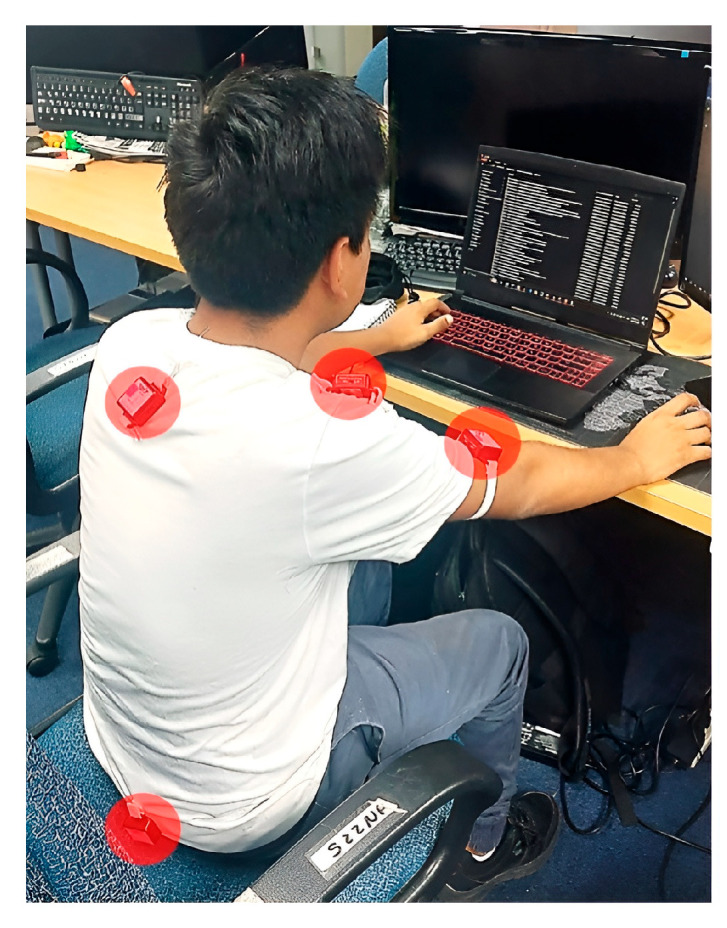
IMU sensor attachment on a participant for upper limb biomechanical assessment.

The sensors were attached to a stretchable, breathable, and form-fitting T-shirt using Velcro^®^ fasteners (Velcro Companies, Agua Prieta, Mexico), ensuring comfort, stability, and firm attachment while allowing quick repositioning, precise anatomical alignment, and reproducible sensor placement across trials, as shown in Figure 27.

A total of 25 healthy volunteers (15 females, 10 males) participated in the study. All participants were aged 20–40 years and right-handed. Written informed consent was obtained from each participant prior to the study, in accordance with the Declaration of Helsinki.

Participants assumed each of the three postural experiments illustrated in Table 10 for 5 min blocks, with a 5 min rest interval between postures.

#### 3.2.2. Injury Risk Assessment Results for the 25 Participants by the FIS

**Figure 28:**
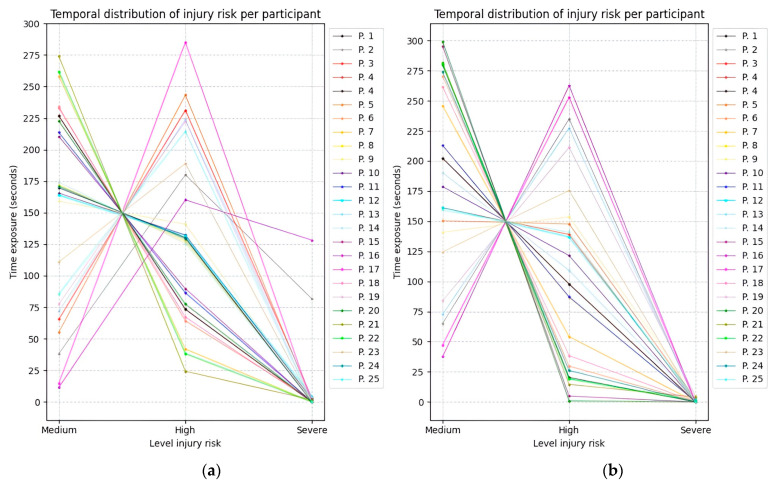
Temporal distribution of upper limb injury risk across twenty-five participants during experiment 1. (**a**) Time exposure for the left upper limb; (**b**) Time exposure for the right upper limb.

**Figure 29:**
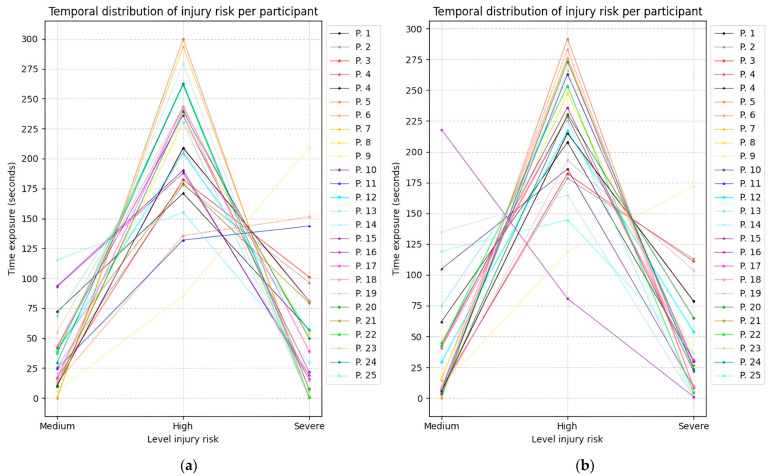
Temporal distribution of upper limb injury risk across twenty-five participants during experiment 2. (**a**) Time exposure for the left upper limb; (**b**) Time exposure for the right upper limb.

**Figure 30:**
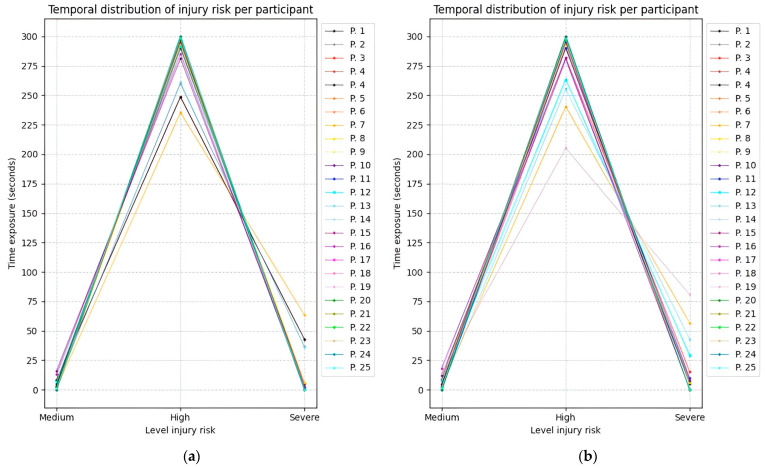
Temporal distribution of upper limb injury risk across twenty-five participants during experiment 3. (**a**) Time exposure for the left upper limb; (**b**) Time exposure for the right upper limb.

Figure 28, Figure 29 and Figure 30 illustrate the temporal evolution of the risk levels for the 25 participants, obtained using the fuzzy inference models while adopting the three proposed postures. The Y-axis represents time, while the X-axis indicates the risk level to which both the right and left sides of the upper limb were exposed.

Based on the time values obtained from Experiment 1, the average duration spent at each risk level is summarized in Table 11. It can be observed that most participants maintained a posture at a medium risk level for more than 2.5 min.

Based on the time values obtained from Experiment 2, the average duration spent at each risk level is presented in Table 12. It can be observed that most participants maintained a posture at a high risk for more than 3.5 min. It is also evident that they spent more time in a posture classified as severe risk compared to the medium-risk level, indicating that this posture corresponds to the highest likelihood of injury.

Based on the time values obtained from Experiment 3, Table 13 summarizes the average duration spent at each risk level. Most participants maintained a high-risk posture for more than 4.5 min.

#### 3.2.3. Injury Risk Assessment Results of 25 Participants by Three Experts Against the FIS

Three experts evaluated each posture observationally using the traditional RULA method in order to compare the results with those obtained by the Fuzzy Inference System (FIS). Table 14 presents the evaluations obtained for four participants. All evaluations are provided in Appendix A, Table A2.

The agreement between the evaluations of the three experts and the computational system was calculated using Fleiss’ Kappa coefficient (see Table 15) according to Table A2. Landis and Koch have characterized different ranges of Kappa values with respect to the degree of agreement among multiple raters. Values greater than 0.75 indicate excellent agreement, values below 0.4 indicate poor agreement, and values between 0.4 and 0.75 indicate moderate to good agreement [53].

The level of agreement between the three expert evaluators and the computational system was classified as good (Landis and Koch [53]) for both sides of the upper limb in the posture of Experiment 1. Similarly, in Experiment 2, agreement between the experts and the system was also considered good for both upper-limb sides. For Experiment 3, the agreement reached a high level according to Landis and Koch for both upper-limb sides.

## 4. Discussion

The results demonstrate that BLE 5.0 substantially enhances the performance of multi-IMU networks compared to BLE 4.0, providing greater range, stability, and scalability. While previous studies typically reported reliable data acquisition with 3 to 4 IMUs, the proposed architecture with six sensors confirms the feasibility of more complex wireless motion capture systems for upper-limb biomechanics. The system achieved transmission ranges of up to 105 m outdoors and 40 m indoors with obstacles, and acquisition efficiency consistently between 99% and 103.7%, with minimal packet loss—even under simultaneous multi-sensor operation at 50 Hz.

Table 16 summarizes representative studies using inertial measurement units (IMUs) for biomechanical and ergonomic motion analysis. While many works reference Bluetooth or BLE technologies, essential communication details—such as the specific Bluetooth version, number of receivers, or data-transmission strategy—are frequently omitted. This lack of standardized reporting limits comparability across studies and obscures the operational constraints of multi-IMU systems. In contrast, the asynchronous communication framework introduced in this work provides deterministic, logically synchronized acquisition across six IMUs, achieving performance comparable to hardware-level synchronization in systems such as Xsens while maintaining full compatibility with open-source software.

Most commercial motion-capture systems referenced in Table 16—such as Xsens, Opal, Noraxon Ultium, Notch, and AnyMo—rely on proprietary software that restricts evaluators’ ability to fully access, manipulate, and visualize data for specialized analyses. In contrast, the open-source communication framework developed in this work, implemented in Python using Bleak and asyncio, enables scalable integration of commercial BLE-based sensors while overcoming architectural constraints typically imposed by SoC manufacturers. This approach substantially increases the flexibility and applicability of commercial IMUs, supporting use cases that extend well beyond the capabilities originally envisioned by their vendors.

This work demonstrates that it is possible to extract IMU data reliably and design an optimized acquisition framework which is capable, in theory, of supporting more sensors than officially specified by the manufacturer while achieving extended communication ranges without additional hardware. Moreover, the use of the cross-platform Bleak library allows data acquisition not only on Windows but also on Linux, enabling future investigations into connectivity performance, latency, and throughput across diverse operating systems.

In addition, this study presents a fuzzy-inference-based adaptation of the Rapid Upper Limb Assessment (RULA) method. By modeling RULA group A and group B components independently and integrating them through a third inference model, the system produces continuous, interpretable risk scores. The fuzzy-logic formulation enables smooth transitions between risk categories, addressing the discrete and coarse resolution of traditional RULA scoring. Case studies with 25 participants performing three seated postures confirmed the system’s ability to consistently discriminate differences in ergonomic risk, including the detection of postures that maintain high or severe risk over prolonged periods.

Although the proposed multi-IMU network performed well in upper-limb biomechanics, future work should extend its evaluation to other anatomical regions and more complex biomechanical applications. Such studies would benefit from increasing the number of sensors, operating at higher sampling frequencies, and incorporating additional variables—including angular velocity and acceleration—beyond the angular-position analysis conducted here.

Overall, this work not only validates the suitability of BLE 5.0 for scalable multi-IMU networks but also advances ergonomic assessment by integrating real-time, multi-sensor acquisition with interpretable fuzzy-inference models. This represents a substantial improvement over existing approaches and opens new avenues for applications in industrial ergonomics, clinical rehabilitation, human–machine interaction, and virtual-reality systems.

## 5. Conclusions

This study presented the design and validation of a wireless network of six IMUs based on the BLE 5.0 standard, integrated through an asynchronous Python communication framework and a LabVIEW interface for real-time visualization and data processing. The proposed architecture demonstrated reliable multi-sensor acquisition, and its performance was rigorously characterized in terms of transmission behavior, temporal stability, and signal integrity.

The principal contributions of this work are:

- A fully implemented wireless multi-IMU architecture leveraging asynchronous BLE 5.0 acquisition, addressing a critical technological gap in scalable upper-limb motion analysis.
- A Python-based communication framework (Bleak + asyncio) seamlessly integrated with LabVIEW for intuitive data handling and user interaction.
- Comprehensive experimental evaluation of key performance metrics—including transmission stability and signal integrity—providing objective evidence rarely reported in comparable multi-sensor studies.
- Demonstration of clinical applicability through a case study using a fuzzy inference system grounded in the Rapid Upper Limb Assessment (RULA), yielding interpretable and explainable outputs that support adoption by physiotherapists and ergonomic-risk evaluators.

Validation was conducted using six IMUs operating at a 50 Hz sampling rate, and the analysis was constrained to angular position variables in accordance with the RULA methodology. Nonetheless, the modular software architecture and underlying technical design are inherently scalable, supporting expansion to larger sensor networks, higher sampling frequencies, and additional biomechanical variables.

Future work will focus on extending the system to applications requiring full tri-axial analysis of IMU data (acceleration, angular velocity, and angular position), strengthening clinical validation across diverse populations, improving multi-sensor synchronization, and integrating the architecture with edge-computing platforms and rehabilitation technologies. These advancements will further enhance the system’s potential for real-world clinical deployment and trustworthy biomechanical monitoring.

## Figures and Tables

**Table 1:** MAC Addresses and GATT UUIDs of Inertial Sensors.

Device	MAC Address	GATT UUID (Write)	GATT UUID (Read)
S1	DA:8C:88:91:05:44	0000ff34-0000-1000-8000-00805f9a34fb	0000ffe9-0000-1000-8000-00805f9a34fb
S2	EF:93:6A:A8:82:CD	0000ff34-0000-1000-8000-00805f9a34fb	0000ffe9-0000-1000-8000-00805f9a34fb
S3	C1:89:73:14:6D:C2	0000ff34-0000-1000-8000-00805f9a34fb	0000ffe9-0000-1000-8000-00805f9a34fb
S4	DC:81:B6:38:AA:06	0000ff34-0000-1000-8000-00805f9a34fb	0000ffe9-0000-1000-8000-00805f9a34fb
S5	FD:E1:B4:D3:0A:AA	0000ff34-0000-1000-8000-00805f9a34fb	0000ffe9-0000-1000-8000-00805f9a34fb
S6	CE:43:07:34:E5:87	0000ff34-0000-1000-8000-00805f9a34fb	0000ffe9-0000-1000-8000-00805f9a34fb

**Table 2:** Comparative performance analysis of BLE 4.0 and BLE 5.0 communication standards.

Parameter	BLE 4.0	BLE 5.0
Technical data transmission speed	1 Mbps	2 Mbps
Net throughput	803 kbps	1.4 Mbps
Maximum indoor range with obstacles	10 m	40 m (Real value measured)
Outdoor line-of-sight range	50 m	105 m (Real value measured)
Compatibility	Only the 4.0 version	With all BLE 5.0 versions, including 4.0

**Table 3:** Theoretical vs. measured BLE throughput per IMU and aggregate (LOS/NLOS, near/far).

Parameter	Line of Sight (LOS)		Non-Line of Sight (NLOS)	
Range	Near	Far	Near	Far
PHY	1 M/L	1 M/LR	1 M/L	1 M/LR
Connection interval	20 ms			
Notification Payload	20 B			
Theoretical per-IMU Throughput	8 kpbs			
Aggregate theoretical (6 IMUs)	48 kbps			
Measured per-IMU Throughput	7.9 kbps	7.6 kbps	7.9 kbps	7.2 kbps

**Table 4:** MAC Address and measured RSSI performance across short and long communication ranges.

Device	MAC Address	RSSI [dBm] (0–1 m)	RSSI [dBm] (2–40 m)
S1	DA:8C:88:91:05:44	(−38, −74)	(−76, −82)
S2	EF:93:6A:A8:82:CD	(−45, −65)	(−82, −94)
S3	C1:89:73:14:6D:C2	(−33, −67)	(−74, −82)
S4	DC:81:B6:38:AA:06	(−29, −59)	(−77, −83)
S5	FD:E1:B4:D3:0A:AA	(−46, −70)	(−83, −91)
S6	CE:43:07:34:E5:87	(−33, −74)	(−77, −83)

**Table 5:** Descriptive statistics for each IMU. Highlighted values indicate the highest and lowest Standard Deviation (SD) and Coefficient of Variation (CV).

Parameter	IMU 1	IMU 2	IMU 3	IMU 4	IMU 5	IMU 6
Mean	14,858.3	14,558.76	15,286.64	15,286.84	15,285.64	15,556.3
Standard Deviation	1209.63	1297.15	751.45	861.19	1014.03	617.47
Variance	1,463,196.24	1,682,600.35	564,673.571	741,656.042	1,028,257.73	381,264.831
Lower value	11,311.5	11,752.5	13,920	11,215.5	11,059.5	14,893.5
Upper value	17,413.5	17,104.5	18,211.5	17,470.5	17,458.5	174,66
Range	6102	5352	4291.5	6255	6399	2572.5
Coefficient of Variation	8.14%	8.91%	4.86%	5.63%	6.63%	3.97%
Bias	−141.7	−441.24	465.86	286.84	286.64	556.3
Mean percentage error	−0.94%	−2.94	3.11%	1.91%	1.91%	3.71%

**Table 6:** Post hoc pairwise comparisons using Dunn’s test.

Device A	Device B	*p*-Uncorrected	*p*-Corrected
S1	S2	0.145702	1
S1	S3	0.000309	0.0046
S1	S4	0.013532	0.2029
S1	S5	0.020086	0.3013
S1	S6	0.000016	0.00025
S2	S3	0.0000005	0.0000081
S2	S4	0.000082	0.00123
S2	S5	0.000189	0.00284
S2	S6	0.000000013	0.000000203
S3	S4	0.177019	1
S3	S5	0.220740	1
S3	S6	0.421936	1
S4	S5	0.998963	1
S4	S6	0.0292	0.43801
S5	S6	0.0510	0.76574

**Table 7:** Statistical summary of data loss (%) across IMUs.

Device	Mean [%]	Median (Quartile 2) [%]	Interquartile Range (IQR) [%]
IMU 1	0.94	−0.09	6.16
IMU 2	4.02	0.56	11.25
IMU 3	−3.10	−1.37	5.46
IMU 4	−1.91	−1.27	4.66
IMU 5	−1.91	−1.41	4.40
IMU 6	−3.70	−2.05	5.94

**Table 8:** Statistical summary of data efficiency (%) across IMUs.

Device	Mean [%]	Median (Quartile 2) [%]	Interquartile Range (IQR) [%]
IMU 1	99.05	100.09	6.16
IMU 2	95.97	99.44	11.25
IMU 3	103.11	101.37	5.46
IMU 4	101.93	101.27	4.56
IMU 5	101.91	101.41	4.40
IMU 6	103.71	102.05	5.94

**Table 9:** Inclusion and exclusion criteria for biomechanical evaluation.

Inclusion Criteria	Exclusion Criteria
Age: 20–40 years Sex: Male/Female Handedness: Right-handed Sedentary occupation: ≥6 h sitting per day Physical health: No acute musculoskeletal pain in the last 48–72 h.	Non-sedentary individuals History of neurological injuries, musculoskeletal or balance disorders. Inability to follow the experimental instructions or maintain sitting posture.

**Table 10:** Postural experiments description and illustrations.

Experiment 1. Upright Posture While Performing Routine Tasks at Their Computer.	Experiment 2. Slouched Posture, as They Would Normally Adopt After Some Time Due to Discomfort or an Inadequate Chair.	Experiment 3. Reclined Posture, with The Back Leaning Backward and Weight Supported on the Lower Back and Cervical Region.
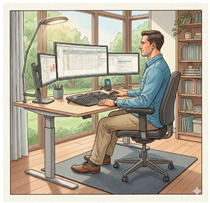	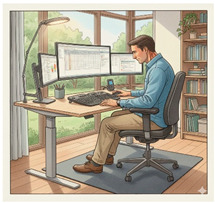	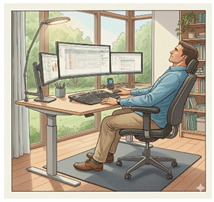

**Table 11:** Average cumulative exposure time (in seconds) per injury risk level for the left and right upper limbs (N = 25) during experiment 1.

Upper Limb Side	Medium Injury Risk [s]	High Injury Risk [s]	Severe Injury Risk [s]
Left	153.6	137.2	9.21
Right	181.15	11.7	0.8

**Table 12:** Average cumulative exposure time (in seconds) per injury risk level for the left and right upper limbs (N = 25) during experiment 2.

Upper Limb Side	Medium Injury Risk [s]	High Injury Risk [s]	Severe Injury Risk [s]
Left	36.19	211.1	52.74
Right	40.42	218.73	40.77

**Table 13:** Average cumulative exposure time (in seconds) per injury risk level for the left and right upper limbs (N = 25) during experiment 3.

Upper Limb Side	Medium Injury Risk [s]	High Injury Risk [s]	Severe Injury Risk [s]
Left	3.1	288.65	8.19
Right	5.23	284.59	10.2

**Table 14:** Assessment of risk levels (Medium—M, High—H, and Severe—S) for four representative participants by each expert evaluator, compared against the FIS.

Participant	Upper Limb Injury Risk Assessment							
	Right Side				Left Side			
	Experiment			Rater	Experiment			Rater
	1	2	3		1	2	3	
1	M	M	H	Expert 1	M	H	H	Expert 1
	M	H	H	Expert 2	M	H	H	Expert 2
	M	H	H	Expert 3	M	H	H	Expert 3
	M	H	H	FIS	M	H	H	FIS
2	H	H	H	Expert 1	H	S	H	Expert 1
	H	H	H	Expert 2	H	H	H	Expert 2
	S	S	H	Expert 3	S	H	H	Expert 3
	H	H	H	FIS	H	H	H	FIS
3	M	H	S	Expert 1	H	H	H	Expert 1
	M	H	H	Expert 2	H	H	H	Expert 2
	M	H	H	Expert 3	H	H	H	Expert 3
	M	H	H	FIS	H	H	H	FIS
4	M	H	H	Expert 1	M	H	H	Expert 1
	M	H	H	Expert 2	M	H	H	Expert 2
	M	H	H	Expert 3	M	H	H	Expert 3
	M	H	H	FIS	M	H	H	FIS

**Table 15:** Kappa coefficient (K) of the agreement level between the three raters and the FIS.

Posture	Right Side	Left Side
1	0.58	0.6
2	0.63	0.57
3	0.75	0.78

**Table 16:** Summary of some recent studies using IMU-based systems with BLE communication for biomechanical analysis.

Author	Year	IMUs	Technology	Frequency	Bluetooth Version
Graham R. [50]	2020	2	HIKOB Fox IMU	100 Hz	-
Tang H. et al. [38]	2021	9	NGIMU BY x-io Technologies	50 Hz	-
Höglund G. et al. [25]	2021	7	MoLab, AnyMo AB	100 Hz	-
Veijalainen P. et al. [3]	2022	1	STMicroelectronics LSM9DS1	50 Hz	5.0
Goreham J. et al. [23]	2022	5	Notch	40 Hz	-
Digo E. et al. [42]	2022	3	Xsens MTx	50 Hz	-
Zhang M. et al. [51]	2022	3	MPU6050	100 Hz	5.0
Sánchez-Fernández L. et al. [6]	2023	6	-	50 Hz	-
Sánchez-Fernández L. et al. [54]	2023	3	-	50 Hz	-
Xiang L. et al. [55]	2024	2	IMeasureU	100 Hz	-
Sánchez-Fernández L. et al. [56]	2024	6	-	50 Hz	-
Razak H. et al. [57]	2025	2	Xsens DOTS + Ultrasound (Gator system)	60 Hz	5.0
J. McNames et al. [58]	2025	6	Opal system	128 Hz	Proprietary ultra-low power 2.4 GHz radio
R. A. Kulkarni et al. [59]	2025	9	Noraxon Ultium Motion	100 Hz	Proprietary wireless protocol

## Data Availability

The data presented in this study are available on request from the corresponding author.

## Abbreviations

The following abbreviations are used in this manuscript:

BLE	Bluetooth Low Energy
IMU	Inertial Measurement Unit
RULA	Rapid Upper Limb Assessment
FIS	Fuzzy Inference System
IoT	Internet of Things
EMG	Electromyography
WHO	World Health Organization
GBD	Global Burden Disease
YLD	Years Live With Disability
AHRS	Attitude and Heading Reference System
MAC	Media Access Control
GATT	Generic Attribute Profile
UUID	Universal Unique Identifier
COM	Communication
RSSI	Received Signal Strength Indicator

## Appendix A. Membership Functions and Some Examples of Rules Designed for the Fuzzy Inference System

Figure A1 shows the complete GUI and control panel for the wireless IMU network.

Figure A2 presents Groups A and B, along with the sequential steps followed by the method to obtain the scores for each group according to the range of motion or the activity performed by the corresponding limbs. The final evaluation of each group is subsequently used to determine the overall risk level to which a person is exposed in occupational environments.

Figure A3 and Figure A4 illustrate the membership functions of the input variables corresponding to the values entered by the evaluator. These values represent the numerical parameters that comprise the upper-limb assessment for Groups A and B, respectively. Their ranges were defined according to the experimental case study, in which the forearms are assumed to be in constant motion, the wrist remains in pronation, and the feet are supported on the ground while seated. Additionally, the wrist may adopt either a neutral or flexed position, and the arms may rest on a support or remain unsupported. These evaluator-defined parameters are entered into the fuzzy inference model, where they are fuzzified together with the variables obtained from the data acquisition process, contributing to the overall ergonomic risk evaluation.

Figure A5 presents the final injury risk assessment levels, according to the total evaluation of Groups A and B.

Table A1 shows the complete rule base for the three fuzzy inference models (A, B, and C).

Table A2 presents the complete risk assessment for 25 participants by three evaluators, compared with the FIS.

**Table A1:** Complete rules of the fuzzy inference models.

Group	Rule	Description
A	1	IF Arm Flexion/Extension is <−20 and Arm Abduction is Low and Supported arms is 0 and Lower arm movement is 3 and Wrist position is 1 and Wrist twist is 2, THEN Numerical output for group A is 4
	2	IF Arm Flexion/Extension is <−20 and Arm Abduction is Medium and Supported arms is 0 and Lower arm movement is 3 and Wrist position is 1 and Wrist twist is 2, THEN Numerical output for group A is 4
	3	IF Arm Flexion/Extension is <−20 and Arm Abduction is High and Supported arms is 0 and Lower arm movement is 3 and Wrist position is 1 and Wrist twist is 2, THEN Numerical output for group A is 4
	4	IF Arm Flexion/Extension is <−20 and Arm Abduction is Low and Supported arms is 1 and Lower arm movement is 3 and Wrist position is 1 and Wrist twist is 2, THEN Numerical output for group A is 3
	5	IF Arm Flexion/Extension is <−20 and Arm Abduction is Medium and Supported arms is 1 and Lower arm movement is 3 and Wrist position is 1 and Wrist twist is 2, THEN Numerical output for group A is 4
	6	IF Arm Flexion/Extension is <−20 and Arm Abduction is High and Supported arms is 1 and Lower arm movement is 3 and Wrist position is 1 and Wrist twist is 2, THEN Numerical output for group A is 4
	7	IF Arm Flexion/Extension is −20 to 20 and Arm Abduction is Low and Supported arms is 0 and Lower arm movement is 3 and Wrist position is 1 and Wrist twist is 2, THEN Numerical output for group A is 3
	8	IF Arm Flexion/Extension is −20 to 20 and Arm Abduction is Medium and Supported arms is 0 and Lower arm movement is 3 and Wrist position is 1 and Wrist twist is 2, THEN Numerical output for group A is 4
	9	IF Arm Flexion/Extension is −20 to 20 and Arm Abduction is High and Supported arms is 0 and Lower arm movement is 3 and Wrist position is 1 and Wrist twist is 2, THEN Numerical output for group A is 4
	10	IF Arm Flexion/Extension is −20 to 20 and Arm Abduction is Medium and Supported arms is 1 and Lower arm movement is 3 and Wrist position is 1 and Wrist twist is 2, THEN Numerical output for group A is 3
	11	IF Arm Flexion/Extension is −20 to 20 and Arm Abduction is High and Supported arms is 1 and Lower arm movement is 3 and Wrist position is 1 and Wrist twist is 2, THEN Numerical output for group A is 3
	12	IF Arm Flexion/Extension is 20 to 45 and Arm Abduction is Low and Supported arms is 0 and Lower arm movement is 3 and Wrist position is 1 and Wrist twist is 2, THEN Numerical output for group A is 4
	13	IF Arm Flexion/Extension is 20 to 45 and Arm Abduction is Medium and Supported arms is 0 and Lower arm movement is 3 and Wrist position is 1 and Wrist twist is 2, THEN Numerical output for group A is 4
	14	IF Arm Flexion/Extension is 20 to 45 and Arm Abduction is High and Supported arms is 0 and Lower arm movement is 3 and Wrist position is 1 and Wrist twist is 2, THEN Numerical output for group A is 4
	15	IF Arm Flexion/Extension is 20 to 45 and Arm Abduction is Low and Supported arms is 1 and Lower arm movement is 3 and Wrist position is 1 and Wrist twist is 2, THEN Numerical output for group A is 3
	16	IF Arm Flexion/Extension is 20 to 45 and Arm Abduction is Medium and Supported arms is 1 and Lower arm movement is 3 and Wrist position is 1 and Wrist twist is 2, THEN Numerical output for group A is 4
	17	IF Arm Flexion/Extension is 20 to 45 and Arm Abduction is High and Supported arms is 1 and Lower arm movement is 3 and Wrist position is 1 and Wrist twist is 2, THEN Numerical output for group A is 4
	18	IF Arm Flexion/Extension is 45 to 90 and Arm Abduction is Low and Supported arms is 0 and Lower arm movement is 3 and Wrist position is 1 and Wrist twist is 2, THEN Numerical output for group A is 4
	19	IF Arm Flexion/Extension is 45 to 90 and Arm Abduction is Medium and Supported arms is 0 and Lower arm movement is 3 and Wrist position is 1 and Wrist twist is 2, THEN Numerical output for group A is 4
	20	IF Arm Flexion/Extension is 45 to 90 and Arm Abduction is High and Supported arms is 0 and Lower arm movement is 3 and Wrist position is 1 and Wrist twist is 2, THEN Numerical output for group A is 4
	21	IF Arm Flexion/Extension is 45 to 90 and Arm Abduction is Low and Supported arms is 1 and Lower arm movement is 3 and Wrist position is 1 and Wrist twist is 2, THEN Numerical output for group A is 4
	22	IF Arm Flexion/Extension is 45 to 90 and Arm Abduction is Medium and Supported arms is 1 and Lower arm movement is 3 and Wrist position is 1 and Wrist twist is 2, THEN Numerical output for group A is 4
	23	IF Arm Flexion/Extension is 45 to 90 and Arm Abduction is High and Supported arms is 1 and Lower arm movement is 3 and Wrist position is 1 and Wrist twist is 2, THEN Numerical output for group A is 4
	24	IF Arm Flexion/Extension is >+90 and Arm Abduction is Low and Supported arms is 0 and Lower arm movement is 3 and Wrist position is 1 and Wrist twist is 2, THEN Numerical output for group A is 4
	25	IF Arm Flexion/Extension is >+90 and Arm Abduction is Medium and Supported arms is 0 and Lower arm movement is 3 and Wrist position is 1 and Wrist twist is 2, THEN Numerical output for group A is 6
	26	IF Arm Flexion/Extension is >+90 and Arm Abduction is High and Supported arms is 0 and Lower arm movement is 3 and Wrist position is 1 and Wrist twist is 2, THEN Numerical output for group A is 6
	27	IF Arm Flexion/Extension is >+90 and Arm Abduction is Low and Supported arms is 1 and Lower arm movement is 3 and Wrist position is 1 and Wrist twist is 2, THEN Numerical output for group A is 4
	28	IF Arm Flexion/Extension is >+90 and Arm Abduction is Medium and Supported arms is 1 and Lower arm movement is 3 and Wrist position is 1 and Wrist twist is 2, THEN Numerical output for group A is 4
	29	IF Arm Flexion/Extension is >+90 and Arm Abduction is High and Supported arms is 1 and Lower arm movement is 3 and Wrist position is 1 and Wrist twist is 2, THEN Numerical output for group A is 4
	30	IF Arm Flexion/Extension is <−20 and Arm Abduction is Low and Supported arms is 0 and Lower arm movement is 3 and Wrist position is 2 and Wrist twist is 2, THEN Numerical output for group A is 4
	31	IF Arm Flexion/Extension is <−20 and Arm Abduction is Medium and Supported arms is 0 and Lower arm movement is 3 and Wrist position is 2 and Wrist twist is 2, THEN Numerical output for group A is 4
	32	IF Arm Flexion/Extension is <−20 and Arm Abduction is High and Supported arms is 0 and Lower arm movement is 3 and Wrist position is 2 and Wrist twist is 2, THEN Numerical output for group A is 4
	33	IF Arm Flexion/Extension is <−20 and Arm Abduction is Low and Supported arms is 1 and Lower arm movement is 3 and Wrist position is 2 and Wrist twist is 2, THEN Numerical output for group A is 3
	34	IF Arm Flexion/Extension is <−20 and Arm Abduction is Medium and Supported arms is 1 and Lower arm movement is 3 and Wrist position is 2 and Wrist twist is 2, THEN Numerical output for group A is 4
	35	IF Arm Flexion/Extension is <−20 and Arm Abduction is High and Supported arms is 1 and Lower arm movement is 3 and Wrist position is 2 and Wrist twist is 2, THEN Numerical output for group A is 4
	36	IF Arm Flexion/Extension is −20 to 20 and Arm Abduction is Low and Supported arms is 0 and Lower arm movement is 3 and Wrist position is 2 and Wrist twist is 2, THEN Numerical output for group A is 3
	37	IF Arm Flexion/Extension is −20 to 20 and Arm Abduction is Medium and Supported arms is 0 and Lower arm movement is 3 and Wrist position is 2 and Wrist twist is 2, THEN Numerical output for group A is 4
	38	IF Arm Flexion/Extension is −20 to 20 and Arm Abduction is High and Supported arms is 0 and Lower arm movement is 3 and Wrist position is 2 and Wrist twist is 2, THEN Numerical output for group A is 4
	39	IF Arm Flexion/Extension is −20 to 20 and Arm Abduction is Medium and Supported arms is 1 and Lower arm movement is 3 and Wrist position is 2 and Wrist twist is 2, THEN Numerical output for group A is 3
	40	IF Arm Flexion/Extension is −20 to 20 and Arm Abduction is High and Supported arms is 1 and Lower arm movement is 3 and Wrist position is 2 and Wrist twist is 2, THEN Numerical output for group A is 5
	41	IF Arm Flexion/Extension is 20 to 45 and Arm Abduction is Low and Supported arms is 0 and Lower arm movement is 3 and Wrist position is 2 and Wrist twist is 2, THEN Numerical output for group A is 4
	42	IF Arm Flexion/Extension is 20 to 45 and Arm Abduction is Medium and Supported arms is 0 and Lower arm movement is 3 and Wrist position is 2 and Wrist twist is 2, THEN Numerical output for group A is 4
	43	IF Arm Flexion/Extension is 20 to 45 and Arm Abduction is High and Supported arms is 0 and Lower arm movement is 3 and Wrist position is 2 and Wrist twist is 2, THEN Numerical output for group A is 4
	44	IF Arm Flexion/Extension is 20 to 45 and Arm Abduction is Low and Supported arms is 1 and Lower arm movement is 3 and Wrist position is 2 and Wrist twist is 2, THEN Numerical output for group A is 3
	45	IF Arm Flexion/Extension is 20 to 45 and Arm Abduction is Medium and Supported arms is 1 and Lower arm movement is 3 and Wrist position is 2 and Wrist twist is 2, THEN Numerical output for group A is 4
	46	IF Arm Flexion/Extension is 20 to 45 and Arm Abduction is High and Supported arms is 1 and Lower arm movement is 3 and Wrist position is 2 and Wrist twist is 2, THEN Numerical output for group A is 4
	47	IF Arm Flexion/Extension is 45 to 90 and Arm Abduction is Low and Supported arms is 0 and Lower arm movement is 3 and Wrist position is 2 and Wrist twist is 2, THEN Numerical output for group A is 4
	48	IF Arm Flexion/Extension is 45 to 90 and Arm Abduction is Medium and Supported arms is 0 and Lower arm movement is 3 and Wrist position is 2 and Wrist twist is 2, THEN Numerical output for group A is 5
	49	IF Arm Flexion/Extension is 45 to 90 and Arm Abduction is High and Supported arms is 0 and Lower arm movement is 3 and Wrist position is 2 and Wrist twist is 2, THEN Numerical output for group A is 5
	50	IF Arm Flexion/Extension is 45 to 90 and Arm Abduction is Low and Supported arms is 1 and Lower arm movement is 3 and Wrist position is 2 and Wrist twist is 2, THEN Numerical output for group A is 4
	51	IF Arm Flexion/Extension is 45 to 90 and Arm Abduction is Medium and Supported arms is 1 and Lower arm movement is 3 and Wrist position is 2 and Wrist twist is 2, THEN Numerical output for group A is 4
	52	IF Arm Flexion/Extension is 45 to 90 and Arm Abduction is High and Supported arms is 1 and Lower arm movement is 3 and Wrist position is 2 and Wrist twist is 2, THEN Numerical output for group A is 4
	53	IF Arm Flexion/Extension is >+90 and Arm Abduction is Low and Supported arms is 0 and Lower arm movement is 3 and Wrist position is 2 and Wrist twist is 2, THEN Numerical output for group A is 5
	54	IF Arm Flexion/Extension is >+90 and Arm Abduction is Medium and Supported arms is 0 and Lower arm movement is 3 and Wrist position is 2 and Wrist twist is 2, THEN Numerical output for group A is 7
	55	IF Arm Flexion/Extension is >+90 and Arm Abduction is High and Supported arms is 0 and Lower arm movement is 3 and Wrist position is 2 and Wrist twist is 2, THEN Numerical output for group A is 7
	56	IF Arm Flexion/Extension is >+90 and Arm Abduction is Low and Supported arms is 1 and Lower arm movement is 3 and Wrist position is 2 and Wrist twist is 2, THEN Numerical output for group A is 4
	57	IF Arm Flexion/Extension is >+90 and Arm Abduction is Medium and Supported arms is 1 and Lower arm movement is 3 and Wrist position is 2 and Wrist twist is 2, THEN Numerical output for group A is 5
	58	IF Arm Flexion/Extension is >+90 and Arm Abduction is High and Supported arms is 1 and Lower arm movement is 3 and Wrist position is 2 and Wrist twist is 2, THEN Numerical output for group A is 5
B	1	IF Neck is 1 and Trunk flexion is Well supported, and Trunk twisting is No twist and Legs is Sitting, THEN Numerical output for group B is 1
	2	IF Neck is 1 and Trunk flexion is 2 to 20, and Trunk twisting is No twist and Legs is Sitting, THEN Numerical output for group B is 2
	3	IF Neck is 1 and Trunk flexion is 20 and 60, and Trunk twisting is No twist and Legs is Sitting, THEN Numerical output for group B is 3
	4	IF Neck is 1 and Trunk flexion is >60, and Trunk twisting is No twist and Legs is Sitting, THEN Numerical output for group B is 5
	5	IF Neck is 1 and Trunk flexion is Well supported, and Trunk twisting is Left twist and Legs is Sitting, THEN Numerical output for group B is 2
	6	IF Neck is 1 and Trunk flexion is 2 to 20, and Trunk twisting is Left twist and Legs is Sitting, THEN Numerical output for group B is 3
	7	IF Neck is 1 and Trunk flexion is 20 and 60, and Trunk twisting is Left twist and Legs is Sitting, THEN Numerical output for group B is 5
	8	IF Neck is 1 and Trunk flexion is >60, and Trunk twisting is Left twist and Legs is Sitting, THEN Numerical output for group B is 6
	9	IF Neck is 1 and Trunk flexion Well supported, and Trunk twisting is Right twist and Legs is Sitting, THEN Numerical output for group B is 2
	10	IF Neck is 1 and Trunk flexion is 2 to 20, and Trunk twisting is Right twist and Legs is Sitting, THEN Numerical output for group B is 3
	11	IF Neck is 1 and Trunk flexion is 20 and 60, and Trunk twisting is Right twist and Legs is Sitting, THEN Numerical output for group B is 5
	12	IF Neck is 1 and Trunk flexion is >60, and Trunk twisting is Right twist and Legs is Sitting, THEN Numerical output for group B is 6
	13	IF Neck is 2 and Trunk flexion is 2 to 20, and Trunk twisting is No twist and Legs is Sitting, THEN Numerical output for group B is 2
	14	IF Neck is 2 and Trunk flexion is 2 to 20, and Trunk twisting is Right twist and Legs is Sitting, THEN Numerical output for group B is 3
	15	IF Neck is 2 and Trunk flexion is 20 and 60, and Trunk twisting is No twist and Legs is Sitting, THEN Numerical output for group B is 4
	16	IF Neck is 2 and Trunk flexion is >60, and Trunk twisting is No twist and Legs is Sitting, THEN Numerical output for group B is 5
	17	IF Neck is 2 and Trunk flexion is Well supported, and Trunk twisting is Left twist and Legs is Sitting, THEN Numerical output for group B is 2
	18	IF Neck is 2 and Trunk flexion is 2 to 20, and Trunk twisting is Left twist and Legs is Sitting, THEN Numerical output for group B is 4
	19	IF Neck is 2 and Trunk flexion 20 and 60, and Trunk twisting is Left twist and Legs is Sitting, THEN Numerical output for group B is 5
	20	IF Neck is 2 and Trunk flexion is >60, and Trunk twisting is Left twist and Legs is Sitting, THEN Numerical output for group B is 6
	21	IF Neck is 2 and Trunk flexion is Well supported, and Trunk twisting is Right twist and Legs is Sitting, THEN Numerical output for group B is 2
	22	IF Neck is 2 and Trunk flexion is 2 to 20, and Trunk twisting is Right twist and Legs is Sitting, THEN Numerical output for group B is 4
	23	IF Neck is 2 and Trunk flexion 20 and 60, and Trunk twisting is Right twist and Legs is Sitting, THEN Numerical output for group B is 5
	24	IF Neck is 2 and Trunk flexion is >60, and Trunk twisting is Right twist and Legs is Sitting, THEN Numerical output for group B is 6
C	1	IF Total score for group A is 4 and Total score for group B is 4, THEN Final injury risk assessment is Medium
	2	IF Total score for group A is 4 and Total score for group B is 5, THEN Final injury risk assessment is High
	3	IF Total score for group A is 5 and Total score for group B is 2, THEN Final injury risk assessment is Medium
	4	IF Total score for group A is 5 and Total score for group B is 3, THEN Final injury risk assessment is Medium
	5	IF Total score for group A is 5 and Total score for group B is 4, THEN Final injury risk assessment is High
	6	IF Total score for group A is 5 and Total score for group B is 6, THEN Final injury risk assessment is Severe
	7	IF Total score for group A is 5 and Total score for group B is 7, THEN Final injury risk assessment is Severe
	8	IF Total score for group A is 6 and Total score for group B is 2, THEN Final injury risk assessment is Medium
	9	IF Total score for group A is 6 and Total score for group B is 3, THEN Final injury risk assessment is High
	10	IF Total score for group A is 6 and Total score for group B is 6, THEN Final injury risk assessment is Severe
	11	IF Total score for group A is 6 and Total score for group B is 7, THEN Final injury risk assessment is Severe
	12	IF Total score for group A is 7 and Total score for group B is 2, THEN Final injury risk assessment is High
	13	IF Total score for group A is 7 and Total score for group B is 5, THEN Final injury risk assessment is Severe
	14	IF Total score for group A is 7 and Total score for group B is 6, THEN Final injury risk assessment is Severe
	15	IF Total score for group A is 7 and Total score for group B is 7, THEN Final injury risk assessment is Severe
	16	IF Total score for group A is 5 and Total score for group B is 5, THEN Final injury risk assessment is High
	17	IF Total score for group A is 6 and Total score for group B is 4, THEN Final injury risk assessment is High
	18	IF Total score for group A is 6 and Total score for group B is 5, THEN Final injury risk assessment is High
	19	IF Total score for group A is 7 and Total score for group B is 3, THEN Final injury risk assessment is High
	20	IF Total score for group A is 7 and Total score for group B is 4, THEN Final injury risk assessment is High
	21	IF Total score for group A is 4 and Total score for group B is 2, THEN Final injury risk assessment is Medium
	22	IF Total score for group A is 4 and Total score for group B is 3, THEN Final injury risk assessment is Medium
	23	IF Total score for group A is 4 and Total score for group B is 6, THEN Final injury risk assessment is High
	24	IF Total score for group A is 4 and Total score for group B is 7, THEN Final injury risk assessment is High

**Table A2:** Risk levels assessment (Medium—M, High—H, Severe—S), indicating evaluation of each expert rater and the FIS across 25 participants.

Participant	Upper Limb Injury Risk Assessment							
	Right Side				Left Side			
	Experiment			Rater	Experiment			Rater
	1	2	3		1	2	3	
1	M	M	H	Expert 1	M	H	H	Expert 1
	M	H	H	Expert 2	M	H	H	Expert 2
	M	H	H	Expert 3	M	H	H	Expert 3
	M	H	H	FIS	M	H	H	FIS
2	H	H	H	Expert 1	H	S	H	Expert 1
	H	H	H	Expert 2	H	H	H	Expert 2
	S	S	H	Expert 3	S	H	H	Expert 3
	H	H	H	FIS	H	H	H	FIS
3	M	H	S	Expert 1	H	H	H	Expert 1
	M	H	H	Expert 2	H	H	H	Expert 2
	M	H	H	Expert 3	H	H	H	Expert 3
	M	H	H	FIS	H	H	H	FIS
4	M	H	H	Expert 1	M	H	H	Expert 1
	M	H	H	Expert 2	M	H	H	Expert 2
	M	H	H	Expert 3	M	H	H	Expert 3
	M	H	H	FIS	M	H	H	FIS
5	M	H	H	Expert 1	H	H	H	Expert 1
	M	H	H	Expert 2	H	H	H	Expert 2
	M	H	H	Expert 3	M	H	H	Expert 3
	M	H	H	FIS	H	H	H	FIS
6	M	H	H	Expert 1	M	S	H	Expert 1
	M	H	H	Expert 2	M	S	H	Expert 2
	M	H	H	Expert 3	M	S	H	Expert 3
	M	H	H	FIS	M	S	H	FIS
7	M	H	H	Expert 1	M	H	H	Expert 1
	M	H	H	Expert 2	M	H	H	Expert 2
	M	H	H	Expert 3	M	H	H	Expert 3
	M	H	H	FIS	M	H	H	FIS
8	M	H	H	Expert 1	M	H	H	Expert 1
	M	H	H	Expert 2	M	H	H	Expert 2
	M	H	H	Expert 3	M	H	H	Expert 3
	M	H	H	FIS	M	H	H	FIS
9	M	S	H	Expert 1	H	H	H	Expert 1
	M	S	H	Expert 2	H	S	H	Expert 2
	H	S	H	Expert 3	M	S	H	Expert 3
	H	S	H	FIS	M	S	H	FIS
10	M	H	H	Expert 1	H	H	M	Expert 1
	M	H	H	Expert 2	M	M	M	Expert 2
	M	H	H	Expert 3	M	H	M	Expert 3
	M	H	H	FIS	M	H	H	FIS
11	M	H	H	Expert 1	M	S	H	Expert 1
	M	H	H	Expert 2	M	S	H	Expert 2
	M	H	H	Expert 3	M	H	H	Expert 3
	M	H	H	FIS	M	S	H	FIS
12	M	H	H	Expert 1	H	H	H	Expert 1
	M	H	H	Expert 2	M	H	H	Expert 2
	M	H	H	Expert 3	M	H	H	Expert 3
	M	H	H	FIS	M	H	H	FIS
13	H	H	H	Expert 1	H	H	H	Expert 1
	H	H	H	Expert 2	H	H	H	Expert 2
	M	H	H	Expert 3	H	H	H	Expert 3
	H	H	H	FIS	H	H	H	FIS
14	M	H	M	Expert 1	M	H	H	Expert 1
	M	H	M	Expert 2	H	H	H	Expert 2
	M	H	M	Expert 3	M	H	H	Expert 3
	M	H	H	FIS	M	H	H	FIS
15	M	H	H	Expert 1	M	H	H	Expert 1
	M	H	H	Expert 2	M	H	H	Expert 2
	M	H	H	Expert 3	M	H	H	Expert 3
	M	H	H	FIS	M	H	H	FIS
16	S	M	S	Expert 1	H	M	H	Expert 1
	H	M	S	Expert 2	S	H	S	Expert 2
	H	M	S	Expert 3	H	H	H	Expert 3
	H	M	H	FIS	H	H	H	FIS
17	H	H	H	Expert 1	H	H	H	Expert 1
	H	H	H	Expert 2	H	H	H	Expert 2
	H	H	H	Expert 3	H	H	H	Expert 3
	H	H	H	FIS	H	H	H	FIS
18	M	H	H	Expert 1	M	H	H	Expert 1
	M	H	H	Expert 2	M	H	H	Expert 2
	M	H	H	Expert 3	M	H	H	Expert 3
	M	H	H	FIS	M	H	H	FIS
19	H	H	M	Expert 1	H	H	H	Expert 1
	H	S	H	Expert 2	H	H	H	Expert 2
	M	H	H	Expert 3	H	H	H	Expert 3
	H	H	H	FIS	H	H	H	FIS
20	M	H	H	Expert 1	M	H	H	Expert 1
	M	H	H	Expert 2	M	H	H	Expert 2
	M	H	H	Expert 3	M	H	H	Expert 3
	M	H	H	FIS	M	H	H	FIS
21	M	H	H	Expert 1	M	H	H	Expert 1
	M	H	H	Expert 2	M	H	H	Expert 2
	M	H	H	Expert 3	M	H	H	Expert 3
	M	H	H	FIS	M	H	H	FIS
22	M	H	H	Expert 1	M	H	H	Expert 1
	M	H	H	Expert 2	M	H	H	Expert 2
	M	H	H	Expert 3	M	H	H	Expert 3
	M	H	H	FIS	M	H	H	FIS
23	M	H	S	Expert 1	M	H	H	Expert 1
	M	H	S	Expert 2	H	H	H	Expert 2
	M	H	S	Expert 3	M	H	H	Expert 3
	H	H	H	FIS	H	H	H	FIS
24	M	H	H	Expert 1	M	H	H	Expert 1
	M	H	H	Expert 2	M	H	H	Expert 2
	M	H	H	Expert 3	H	H	H	Expert 3
	M	H	H	FIS	M	H	H	FIS
25	M	H	H	Expert 1	H	M	H	Expert 1
	M	H	H	Expert 2	H	M	H	Expert 2
	M	M	H	Expert 3	H	M	H	Expert 3
	M	H	H	FIS	H	H	H	FIS
